# Photoreactivity of Norrish Type Photoinitiators for 3D Laser Printing via First Principles Calculations

**DOI:** 10.1002/marc.202500231

**Published:** 2025-05-15

**Authors:** Anna Mauri, Pascal Kiefer, Wolfgang Wenzel, Mariana Kozlowska

**Affiliations:** ^1^ Institute of Nanotechnology (INT) Karlsruhe Institute of Technology (KIT) Kaiserstraße 12 76131 Karlsruhe Germany; ^2^ Institute of Applied Physics (APH) Karlsruhe Institute of Technology (KIT) Kaiserstraße 12 76131 Karlsruhe Germany

**Keywords:** 3D laser printing, density functional theory, free‐radical polymerization, photoinitiator, two‐photon polymerization

## Abstract

In the rapidly evolving field of 3D laser nanoprinting, achieving high resolution and high printing speed relies heavily on the effective use and design of sensitive photoinitiators. However, their photoreactivity is hitherto not well described. This study investigates the photochemical and photophysical characteristics of a high‐performing Norrish Type II photoinitiator (2*E*,6*E*)‐2,6‐bis(4‐(dibutylamino)benzylidene)‐4‐methylcyclohexanone, known as BBK, as well as commonly employed Norrish Type I photoinitiators: Irgacure 651, Irgacure 369. Using quantum mechanical calculations, multiphoton absorption, the formation of the excited states, and the radical formation mechanisms most probably involved in the initiation of free radical polymerization are examined. Their relation to the observations during 3D printing experiments is discussed, aiming to uncover the molecular foundations behind varying performances of photoinitiators. Bond dissociation energies and energy barriers for bond cleavage of Irgacure photoinitiators are demonstrated to confirm radical formation in the lowest triplet state, whereas this pathway is shown to be less probable for BBK. The radical polymerization initiation upon absorption from the triplet manifold of BBK and reactions with pentaerythritol triacrylate (PETA) monomers are described. Deactivation pathway via reversible intersystem crossing, as well as the photoactivation characteristics, are compared with relation to the 7‐diethylamino‐3‐thenoylcoumarin (DETC) photoinitiator.

## Graphical abstract

Quantum mechanical calculations demonstrate that Norrish Type I photoinitiators generate radicals in 3D laser printing through two‐photon absorption and bond cleavage in the lowest triplet state. In contrast, Type II photoinitiators initiate polymerization via processes involving higher triplet states and a unique third‐order mechanism. This pathway enhances resolution in 3D laser printing.

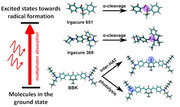

## 1 Introduction

Photoinitiators (PIs) absorb photons to activate a polymerization reaction by forming reactive species such as anions, cations, or radicals.^[^ ^1^ ^]^ They are fundamental in forming the polymers that are integral to many aspects of modern life.^[^ ^2^ ^]^ The nature of the reactive species formed divides PIs into cationic (anionic) or radical types.^[^ ^1^, ^3^ ^]^ Cationic PIs, such as iodonium and sulfonium salts, generate Lewis or Brønsted acids that initiate polymerization, while radical PIs produces highly reactive species with unpaired electrons.^[^ ^3^, ^4^ ^]^

**Figure 1:**
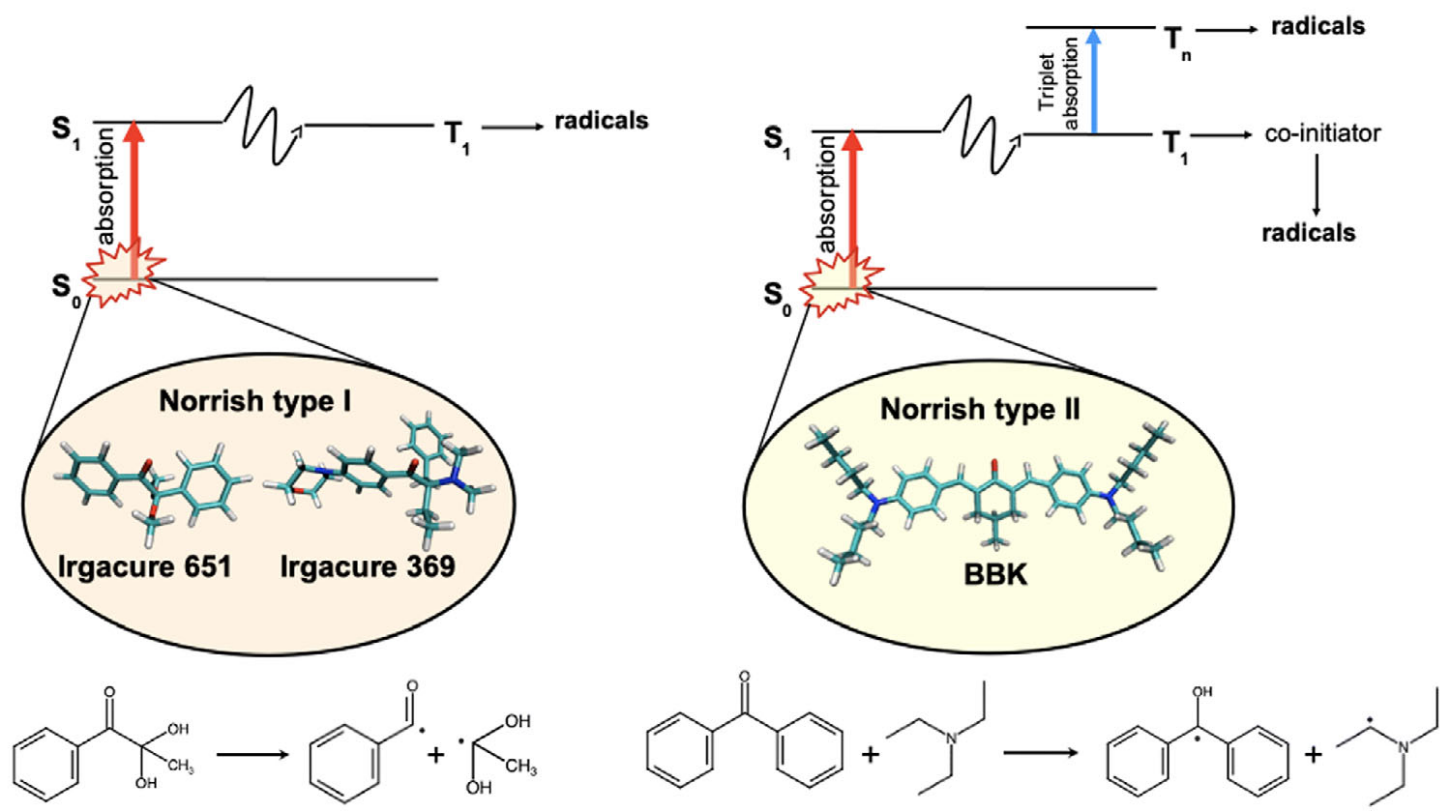
Radical formation mechanisms for Norrish Type I, e.g., Irgacure 651 and Irgacure 369 (left), and type II PIs, e.g., BBK (right). The formation of radicals occurs through a unimolecular (type I) or bimolecular (type II) process as a result of the absorption from the ground state to the singlet excited state and subsequent ISC to the triplet state. For Norrish Type‐II PIs, the additional triplet‐triplet absorption (TA) channel contributing to the formation of high triplet excited states capable of generating radicals is also depicted. PI molecules studied in this work are highlighted for clarity.

PIs have gained popularity in a rapidly growing technology: 3D laser nanoprinting, allowing for a two‐photon polymerization (TPP) for high‐resolution fabrication in the nanometer range.^[^ ^5^, ^6^, ^7^, ^8^, ^9^ ^]^ In this process, PIs are known to create active species in triplet states through a previous multiphoton absorption, e.g., two‐photon absorption (2PA) from the ground state to the singlet manifold followed by intersystem crossing (ISC) to the triplet states, ultimately forming a stable, cross‐linked polymer network.^[^ ^6^, ^7^ ^]^ Within the category of 2PA‐based free radical initiators, Norrish Type I and type II^[^ ^10^ ^]^ are commonly used. Although both types of PIs absorb light, their mechanisms for generating radicals during TPP differ, affecting their reactivity and selectivity (as illustrated in **Figure** **1**). Norrish type I PIs, e.g., hydroxy ketones, α‐aminoalkylphenones, generally undergo bond cleavage upon absorption, yielding two radical fragments that initiate polymerization.^[^ ^3^, ^11^, ^12^ ^]^ Common fragmentation mechanisms for this PI type include α‐ and β‐cleavage: the former involves homolytic scission adjacent to the carbonyl group of the initiator, producing highly reactive radical fragments, while β‐cleavage occurs generally at other weak bonds that are not necessarily associated with the carbonyl moiety.^[^ ^13^ ^]^ Heteroatoms can indeed weaken nearby bonds (e.g., C–Cl, C–S, or C–N), allowing for potential β‐cleavage and different radicals to form. Norrish Type II PIs (e.g., benzophenones),^[^ ^3^, ^11^, ^12^, ^14^, ^15^ ^]^ on the other hand, typically undergo reactions like intermolecular hydrogen atom transfer (HAT)^[^ ^13^, ^16^, ^17^ ^]^ or electron transfer (ET)^[^ ^3^, ^18^ ^]^ involving another molecular unit, which is called a co‐initiator, such as an amine for HAT or a salt for ET, to create a highly reactive state. The process is typically defined as bimolecular for this reason. Amine co‐initiators are often used as they show good hydrogen atom donor capabilities,^[^ ^13^, ^16^, ^17^ ^]^ allowing for the radical formation, thus polymerization initiation (see Figure 1). Finally, the unsaturated double C = C bond of the monomer is converted into a single C–C bond^[^ ^19^, ^20^, ^21^, ^22^, ^23^ ^]^ triggering a chain reaction that results in the formation of the polymer network. Different types of radicals can activate the free radical polymerization (FRP) of monomers depending on the radical formation channels.^[^ ^20^ ^]^

Although TPP with the use of Norrish Type PIs has advanced 3D laser nanoprinting, challenges remain in improving resolution and printing speed due to diffraction limits and voxel size constraints.^[^ ^5^, ^6^, ^19^, ^24^, ^25^, ^26^ ^]^ Stimulated emission depletion (STED) offers a potential solution by refining polymerization control through a depletion laser, yet its implementation in 3D printing is still scarce, with limited suitable PIs and power constraints.^[^ ^8^ ^]^ Another approach is the use of sensitive photoresists which effectively allow for very low polymerization threshold powers and faster 3D laser printing, however, there are not many examples of such PIs for technological applications.^[^ ^26^ ^]^ A recently introduced competing process to 2PA in polymerization reactions for 3D laser printing is two‐step absorption, which uses low power continuous wave laser sources.^[^ ^25^, ^27^, ^28^ ^]^ However, this process is currently not fully understood theoretically.^[^ ^27^, ^29^, ^30^ ^]^

In this manuscript, we focus on the radical initiation mechanisms of different Norrish Type I and Type II PIs. For the first category, we have selected Irgacure 369, which is a frequently used initiator for TPP fabrication of structures for different applications. When combined with the monomer PETA, it is characterized by an α‐cleavage fragmentation reaction, leading to the formation of a benzoyl radical and an alkylamino radical generated from the first triplet state.^[^ ^31^, ^32^, ^33^ ^]^ It was also used in microfabrication and 3D laser printing of SZ2080‐mixtures in combination with other PIs.^[^ ^26^ ^]^ Since Irgacure 651 is another highly efficient PI in the Norrish Type I group, which was reported for the α‐cleavage of the compound into a benzoyl and dimethoxybenzyl radicals when combined with poly(methyl methacrylate) (PMMA)^[^ ^34^ ^]^ and shows 2PA characteristics when irradiated with the light wavelengths in the range of 500–600 nm,^[^ ^35^ ^]^ it was also studied. No other molecules are involved in the activation of such photoresists (e.g., co‐initiators), thus the radicals should be formed exclusively upon absorption by such photoinitiators^[^ ^6^, ^26^, ^36^, ^37^, ^38^, ^39^ ^]^ (see the scheme in Figure 1). For the category of Norrish Type II PIs, we have chosen BBK, recognized recently as one of the most promising and sensitive type II PIs reported in combination with, for example, the PETA monomer ^[^ ^26^, ^37^, ^40^ ^]^ or monomer mixture IP‐DIP NPI.^[^ ^41^ ^]^ It demonstrates a surprisingly effective nonlinear behavior (estimated using the “reciprocity law” for N‐photon absorption) indicative of a third‐order process, akin to what has been observed for DETC in the absence of co‐initiators.^[^ ^7^, ^33^ ^]^ The in‐depth study of DETC has been reported recently.^[^ ^19^ ^]^ 3D printing with Irgacure‐type PIs, including Irgacure 369, is known to equal to two instead, i.e., N = 2, which occurs upon absorption of two photons.

Furthermore, BBK has been observed to outperform DETC in terms of printing efficiency.^[^ ^26^, ^41^ ^]^ It allowed to decrease the polymerization threshold power even using lower concentrations than in comparison to the DETC‐containing photoresist.^[^ ^42^ ^]^ Moreover, the substitution of the N‐methyl groups by butyl groups in BBK in comparison to the parent cyclohexanone‐based initiator ((2*E*,6*E*)‐2,6‐bis(4‐(dimethylamino)benzylidene)‐4‐methylcyclohexanone)^[^ ^43^ ^]^ enabled the solubility increase, permitting several times lower polymerization threshold power using BBK.^[^ ^26^, ^42^ ^]^ A reduction in the printing threshold has been noted in several studies conducted on BBK using a 800 nm fs laser alongside a second continuous‐wave (CW) laser at either 640 or 532 nm.^[^ ^37^ ^]^ In addition, the inhibition of BBK with 800 nm CW, but DETC with fs laser wavelength of 800 nm using an 80 MHz printing, tuning the printing resolution, was reported.^[^ ^37^ ^]^ Diverse experimental observations and the summary of PIs for 3D printing^[^ ^26^ ^]^ indicate that differences in the chemical composition of PIs (even of the same class) modulate the photochemical activation of FRP. Since there is still a gap between the molecular understanding of PIs and reasons for their different performance far beyond their 2PA cross‐sections, more investigations are necessary. To integrate this new puzzle piece into the complex landscape of TPP‐based 3D printing, this work elucidates the reaction mechanisms and photoreactivity of Norrish Type I and Norrish Type II PIs, highlighting their similarities and differences, hoping to give new hints for the 3D printing improvements.

## 2 Results and Discussion

The calculated absorption spectra for Irgacure 651, Irgacure 369, and BBK, were computed to explore their photochemical characteristics and reactivity (see Tables S1–S3, Supporting Information). The selection of functionals used in this study derives from an in‐depth comparison between calculated absorption spectra and available experimental data, a well‐established practice in the field^[^ ^20^, ^44^ ^]^ (see the explanation in Supporting Information). Additionally, charge transfer properties are analyzed using the corrected linear response (cLR) approach^[^ ^45^ ^]^ to account for solvent dynamical changes, as extensively discussed in the case of DETC.^[^ ^19^ ^]^ Based on this evaluation, the CAM‐B3LYP functional was applied for Irgacure 651 and Irgacure 369, while both B3LYP and CAM‐B3LYP were employed for BBK (refer to Sections S2 and S3, Supporting Information, for further details).

### 2.1 Multiphoton Absorption Properties

The 1PA spectrum for Irgacure 651 shows an absorption peak at 328.5 nm (see Table S1, Supporting Information), 13 nm blue shifted with respect to the experimental maximum reported value of 341 nm.^[^ ^34^ ^]^ The experimental absorption of this initiator spans in the range 250‐341 nm, depending on the concentration.^[^ ^34^, ^35^ ^]^ The absorption band of Irgacure 369 (reported in Table S2, Supporting Information) shows a hypsochromic shift of approximately 17 nm when compared to experimental data measured in methanol (303.5 nm in contrast with 324 nm^[^ ^35^, ^46^ ^]^). It should be considered that this PI shows a concentration dependence with a peak absorption between 200 nm and approximately 425 nm.^[^ ^46^ ^]^ The 1PA spectrum for BBK in implicit acetonitrile (ACN) using B3LYP presents a red shift of approximately 19 nm when compared to experimental results (see Table S3, Supporting Information): 467.7 nm in theory versus 448 nm in experiment. The peak maximum in toluene was reported to be at 435 nm^[^ ^40^ ^]^ and it also shows a concentration dependence.^[^ ^47^ ^]^ Calculations with CAM‐B3LYP result in a blue shift of around 70 nm (377.6 nm) from experimental data in ACN, similar to the behavior observed for DETC.^[^ ^19^ ^]^

**Figure 2:**
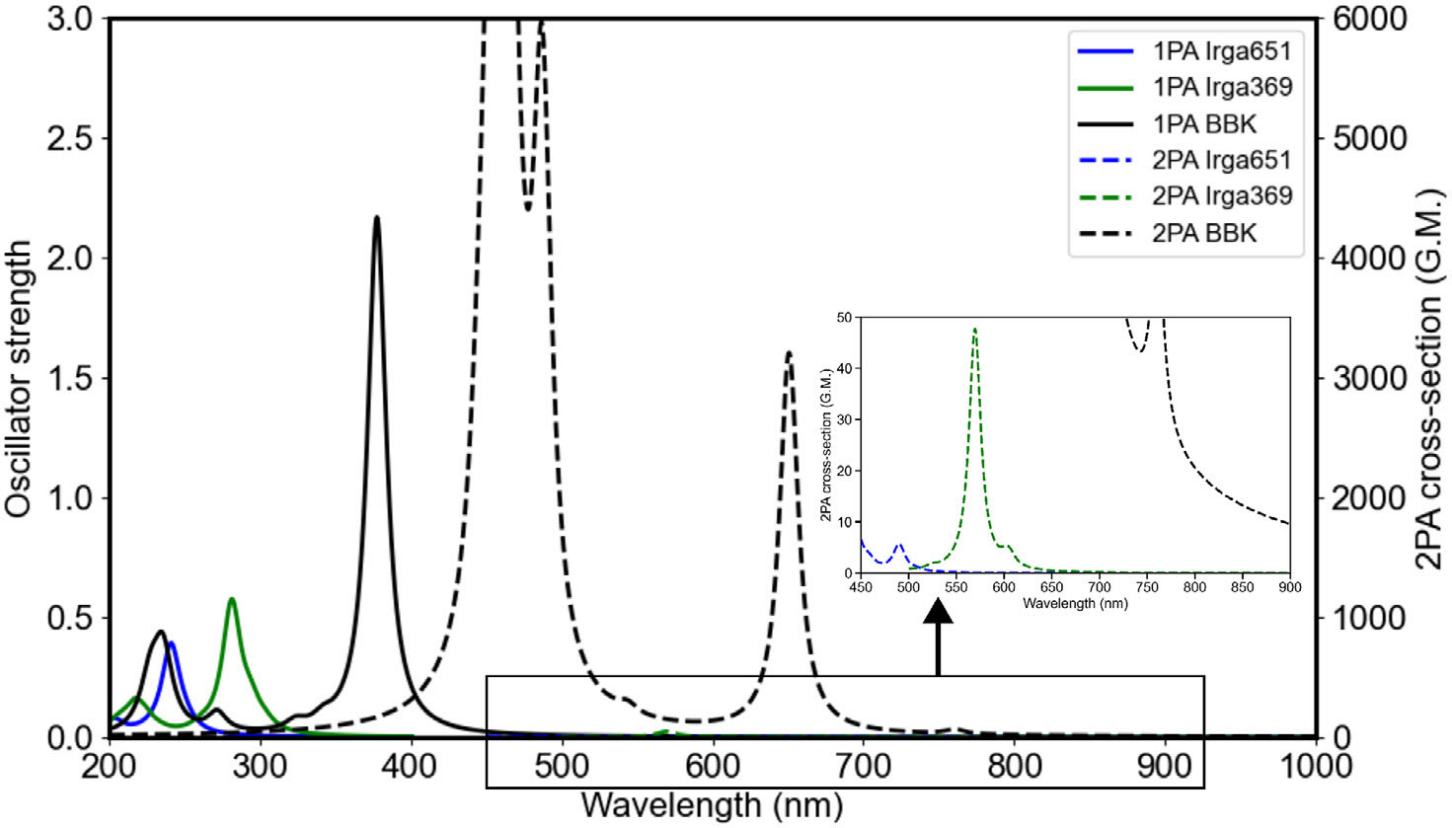
Computed 2PA of Irgacure 651 (labeled as Irga651), Irgacure 369 (labeled as Irga369) and BBK using TD‐CAM‐B3LYP‐D3(BJ)/def2‐TZVP in implicit ACN. The respective peak positions and intensities are given in Tables S16–S18 (Supporting Information). Spectra were plotted as reported in Computational Details section. Experimentally measured values were obtained at ∼ 500 nm,^[^ ^38^ ^]^ ∼ 650–800 nm^[^ ^6^, ^9^, ^48^ ^]^ for Irgacure 651 and Irgacure 369, respectively, and ∼ 800 nm for BBK.^[^ ^44^, ^49^ ^]^

The 2PA spectra for Norrish Type I PIs, depicted in **Figure** **2**, reveal distinct absorption peaks at 502.98 and 609.3 nm, with corresponding cross‐sections of 0.5 and 2.9 GM for Irgacure 651 and Irgacure 369, respectively (see Tables S16 and S17, Supporting Information). Reported values in literature for Irgacure 369 describe 2PA activity around 800 nm^[^ ^6^, ^9^, ^35^ ^]^ when the light intensity reaches a certain threshold,^[^ ^35^, ^43^ ^]^ while 2PA for Irgacure 651 is reported at shorter wavelengths.^[^ ^35^, ^38^ ^]^ In addition, it is extensively reported that the 2PA cross‐sections for Norrish Type I PIs are below 30 GM^[^ ^6^, ^9^, ^35^ ^]^ i.e., 28 GM at around 500 nm for Irgacure 651, while 7 GM at 650 nm and 0.3 GM at 800 nm for Irgacure 369.^[^ ^9^, ^35^ ^]^ The computed 2PA spectrum for Irgacure 651 shows absorption between 484 and 656 nm with cross‐section values ranging from 8 to 0.1 GM, as expected for such a PI. The 2PA spectrum for Irgacure 369 displays a blue shift relative to the experimentally used wavelengths near 800 nm (max at 609 nm with 2.9 GM). The shift is likely due to the DFT functional used in the calculations, which also results in a blue shift of approximately 20 nm for its one‐photon absorption spectrum, as well as the lack of explicit environmental and vibrational effects in the calculated 2PA.

BBK belongs to the benzylidene‐based initiators category. The molecule of a similar structure, but possessing dimethylamino modification ((2*E*,6*E*)‐2,6‐bis(4‐(dimethylamino)benzylidene)‐4‐methylcyclohexanone) instead of dibuthylamino in BBK was reported to show a 2PA cross‐section of 191 GM in dichloromethane obtained using Z‐scan.^[^ ^43^, ^50^ ^]^ It is higher than the values reported for DETC, ranging between 40 and 80 GM at approximately 800 nm.^[^ ^44^, ^49^ ^]^ The calculated 2PA spectrum of BBK in ACN presents a peak corresponding to the S_0_ → S_1_ transition at 760.6 nm, with a cross‐section of 40.5 GM when using the CAM‐B3LYP functional (see Figure 2; Table S18, Supporting Information). For comparison, the computed spectrum for DETC shows the highest peak at 720.84 nm with a cross‐section of 119 GM (see Figure S13 in Ref. [19]). It may be connected to the lower charge‐transfer character of the first singlet excited state of BBK, explained in the Supporting Information. However, there is a significant difference between 2PA cross‐section involving higher‐lying excited states with values even around 3000 GM for BBK (see Tables S18 and S19, Supporting Information). This was not observed for DETC. When the B3LYP functional is used (Table S19, Supporting Information), the cross‐section for BBK is estimated to be even stronger, i.e., 8820 GM at 807.7 nm, however, it is connected to the second 2PA excitation (i.e., a different excited state may be reached) and the accuracy of B3LYP for 2PA is typically lower.^[^ ^51^, ^52^ ^]^ Since vibrational and solvent effects tend to modulate 2PA response of photoactive molecules,^[^ ^53^, ^54^ ^]^ a more advanced analysis and experimental measurements would be necessary for a conclusive comparison. Generally, the 2PA differences of benzylidene ketone‐based initiators were attributed to structural factors such as the nonplanarity of the six‐membered ring and ring substitutions.^[^ ^43^ ^]^ It should be noted that differences in cross‐sections reported in experiments and theory may stem from the differences in the experimental setup and referencing systems. Three‐photon excitations of BBK are reported in Tables S20 and S21 (Supporting Information).

### 2.2 Photophysical Rates Between Singlet and Triplet States

The mechanism of the formation of the T_1_ state for Norrish Type I and type II PIs is similar: with 2PA, the S_1_ state can be reached, and after the ISC, the triplet manifold is obtained. The S_1_ state of Irgacure 651 and Irgacure 369 at 3.43 and 3.27 eV, respectively (see **Table** **1**; Figure S12, Supporting Information), possesses *n*π* character, as reported in Figure S7 (Supporting Information). The ISC to T_1_ (see Figure S7) is El‐Sayed allowed for 46% and 70%.^[^ ^55^, ^56^, ^57^ ^]^

**Table 1:** Adiabatic energies for Irgacure 651 and Irgacure 369. Excited states were optimized with TD‐CAM‐B3LYP‐D3(BJ)/def2‐TZVP in implicit ACN. Adiabatic energy values are expressed in eV and include the zero point energy correction (ZPVE). The Jablonski diagram of the intiators is reported in Figure S12 (Supporting Information).

Photoinitiator	State	Energy
Irgacure 651	S_1_	3.43
	T_1_	2.62
Irgacure 369	S_1_	3.27
	T_1_	2.46

As reported in **Table** **2**, the ISC rates in ACN for Irgacure 651 and Irgacure 369 are both in the order of 10^7^ s^−1^ with similar spin‐orbit coupling (SOC) (1.6 cm^−1^ and 1.2 cm^−1^, respectively, as reported in Table S22, Supporting Information). The ISC rates are competitive with the non‐radiative decay (internal conversion, IC) and are higher than the radiative decay (fluorescence) rates. The weak fluorescence observed for Norrish Type I PIs is in agreement with experimental data from Fischer00A0;al.^[^ ^25^ ^]^

**Table 2:** Most important photophysical rates (in s^−1^) for Irgacure 651 and Irgacure 369 computed with TD‐CAM‐B3LYP‐D3(BJ)/def2‐TZVP in implicit ACN. All photophysical rates are reported in Table S22 (Supporting Information).

Photoinitiator	Fluorescence	IC S_1_‐S_0_	ISC S_1_‐T_1_	Phosphorescence
Irgacure 651	4.53 × 10^5^	1.35 × 10^7^	4.54 × 10^7^	7.93 × 10^−1^
Irgacure 369	4.21 × 10^2^	1.37 × 10^7^	7.92 × 10^7^	3.96 × 10^−1^

From the S_1_ state of BBK, reached upon 2PA, the ISC S_1_‐T_3_ of 2.1 × 10^6^ s^−1^ with SOC of 3.6 cm^−1^ and Δ*E*_S‐T_ of ‐0.1 eV (Table S23 and Figure S13, Supporting Information) is the most probable, allowed by 76% (ππ‐*n*π). Even if SOC is more than twice higher than for the Norrish Type I PIs, the ISC is lower. In addition, the calculated fluorescence and internal conversion rates with the CAM‐B3LYP functional are higher, however, the explicit solvent environment was not considered in calculations. The fluorescence rate is in the same order of magnitude as that of DETC.^[^ ^19^ ^]^ ISC to higher triplet states could not be estimated due to high instability during the optimization process. Therefore, only a limited number of excited states could be successfully optimized with CAM‐B3LYP. For this reason, the B3LYP functional was also employed (see **Table** **4**; Table S24, Supporting Information). Values for all rates are rather consistent, with some differences in ISC. The ISC rate S_1_‐T_2_ is 40% allowed and amounts to 3.7 × 10^8^ s^−1^ with SOC of 3.4 cm^−1^ and Δ*E*_S‐T_ of 0.3 eV (Figure S13, Supporting Information). Thus, it indicates a higher probability of the triplet manifold formation. It should be considered that the TD‐DFT approach has limitations in capturing subtle differences in CT states, especially in higher excited states, but higher level methods are not feasible for similar calculations.

**Table 3:** Adiabatic energies of the excited states of BBK optimized with TD‐CAM‐B3LYP‐D3(BJ)/def2‐TZVP and TD‐B3LYP‐D3(BJ)/def2‐TZVP in implicit ACN. Adiabatic energy values are expressed in eV and include the zero point energy correction (ZPVE). The Jablonski diagram of the initiator is reported in Figure S13 (Supporting Information).

Functional	S_1_	T_1_	T_2_	T_3_	T_4_	T_5_	T_6_	T_7_
CAM‐B3LYP	2.87	1.96	2.44	2.96				
B3LYP	2.35	1.79	2.04	2.61	2.80	3.10	3.17	3.32

**Table 4:** The most important photophysical rates (in s^−1^) for BBK computed with TD‐CAM‐B3LYP‐D3(BJ)/def2‐TZVP in implicit ACN. All photophysical rates are reported in Table S23 (Supporting Information).

Functional	Fluorescence	IC S_1_‐S_0_	ISC S_1_‐T_2_	ISC S_1_‐T_3_	Phosphorescence
CAM‐B3LYP	6.72 × 10^8^	1.38 × 10^8^	1.09 × 10^5^	2.12 × 10^6^	9.41 × 10^−1^
B3LYP	4.15 × 10^8^	1.49 × 10^8^	3.70 × 10^8^	5.14 × 10^6^	1.15 × 10^0^

### 2.3 Cleavage Reaction for Norrish Type I PIs in the Triplet State

**Figure 3:**
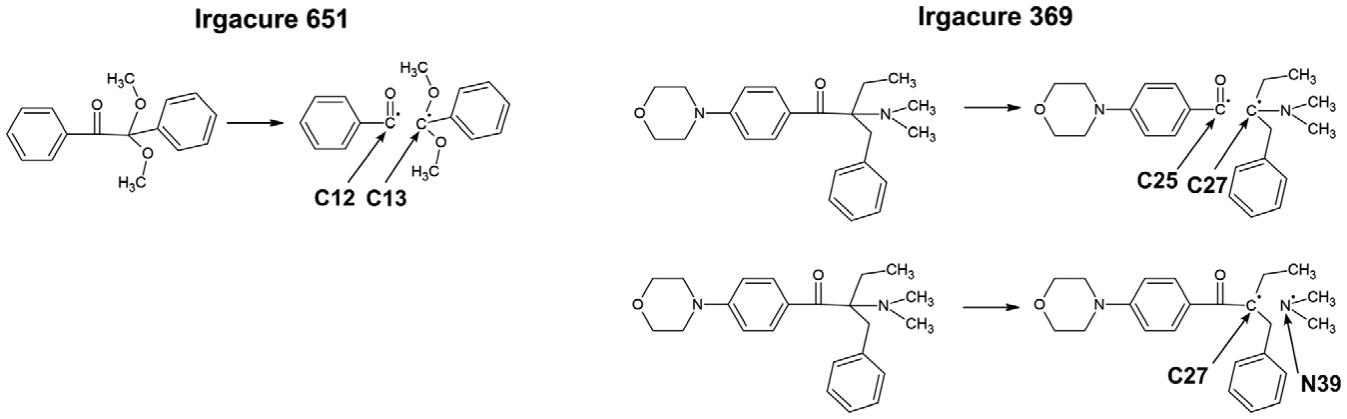
Fragmentation mechanisms for Irgacure 651 (α‐cleavage) and Irgacure 369 (α‐ and β‐cleavage). Bond dissociation energies starting from S_0_ and T_1_ with involved atoms are reported in Table 5.

The mainly reported fragmentation mechanism of Irgacure 651 is the α‐cleavage,^[^ ^34^, ^58^ ^]^ which generates benzoyl and α, α‐dimethoxybenzyl radicals upon light absorption (see **Figure** **3**). Meanwhile, although both α‐ and β‐cleavage mechanisms are known for Irgacure 369,^[^ ^32^ ^]^ with the generation of benzoyl and alkyl radicals or α‐alkyl and α‐alkylamino radicals, respectively, the most common reported reaction for this PI is the α‐cleavage.^[^ ^32^, ^59^ ^]^ The involved bonds are C12–C13 for Irgacure 651, C25–C27 (α‐cleavage) and C27–N39 (see Figure 3; Figure S1, Supporting Information) (β‐cleavage) for Irgacure 369, which are listed in **Table** **5** and reported in Figure 3 and Figure S1 (Supporting Information).

**Table 5:** BDEs (in kcal mol^−1^) computed in CAM‐B3LYP‐D3(BJ)/def2‐TZVP and B3LYP‐D3(BJ)/def2‐TZVP in ACN related to all the possible fragmentation reactions of Irgacure 651 and Irgacure 369. The energy of T_1_ for Irgacure 651 is 60.3 kcal mol^−1^ with both functionals, for Irgacure 369 is 56.6 kcal mol^−1^ (CAM‐B3LYP) and 55.7 kcal mol^−1^ (B3LYP), see Table S25.

	Irgacure 651			Irgacure 369			
Functional	Type of cleavage	Bond	S_0_	T_1_	Atoms	S_0_	T_1_
CAM‐B3LYP	α‐	C12‐C13	55.20	−4.59	C25‐C27	57.46	0.64
	β‐	−	−	−	C27‐N39	47.85	−8.97
B3LYP	α‐	C12‐C13	57.39	−3.28	C25‐C27	56.03	0.18
	β‐	−	−	−	C27‐N39	46.25	−9.60

The dissociation mechanism of Norrish Type I PIs in the triplet state (T_1_) was explored by computing bond dissociation energies (BDEs), which allows to define the energy necessary to break a certain bond. The calculated BDEs related to the dissociation reaction for both initiators were computed with both CAM‐B3LYP and B3LYP functional, for comparison purposes, and reported in Table 5 and Table S25 (Supporting Information). Data suggest that bond cleavage is energetically more favorable when the respective PI is in T_1_, with values of –4.6 kcal mol^−1^ and 0.6 kcal mol^−1^ for Irgacure 651 and Irgacure 369, respectively. Such fragmentation reaction cannot take place when the initiators are in the ground state, where the BDEs are 55.2 and 57.5 kcal mol^−1^, respectively.

As reported in Refs.[27, 60, 61], the cleavage of bonds of Norrish Type I PIs in the T_1_ state is likely to occur if the BDE of the ground state structure is lower than the optimized energy of T_1_, i.e., $E_{S_{0}}^{\text{BDE}}$ < $E_{T_{1}}$. Results listed in Table 1 and Table 5 indicate that for Irgacure 651 the T_1_ energy is higher than the BDE of the ground state (60.3 vs 55.2 kcal mol^−1^). For Irgacure 369, the T_1_ energy is higher than the bond energy in the case of β‐cleavage (56.6 vs 47.9 kcal mol^−1^), but the values are rather similar in the case of α‐cleavage with CAM‐B3LYP (56.6 vs 57.5 kcal mol^−1^). However, considering that the almost negligible difference between the T_1_ energy and the bond energy in the latter case (less than 1 kcal mol^−1^) is in the range of error and that the comparison is functional dependent (see results in B3LYP in Table **3**), the general rule applied for the reluctant Norrish Type I PIs is well confirmed here. It is clearly visible that the dissociation reaction for Irgacure 651 and Irgacure 369 will occur in the lowest triplet state.

**Figure 4:**
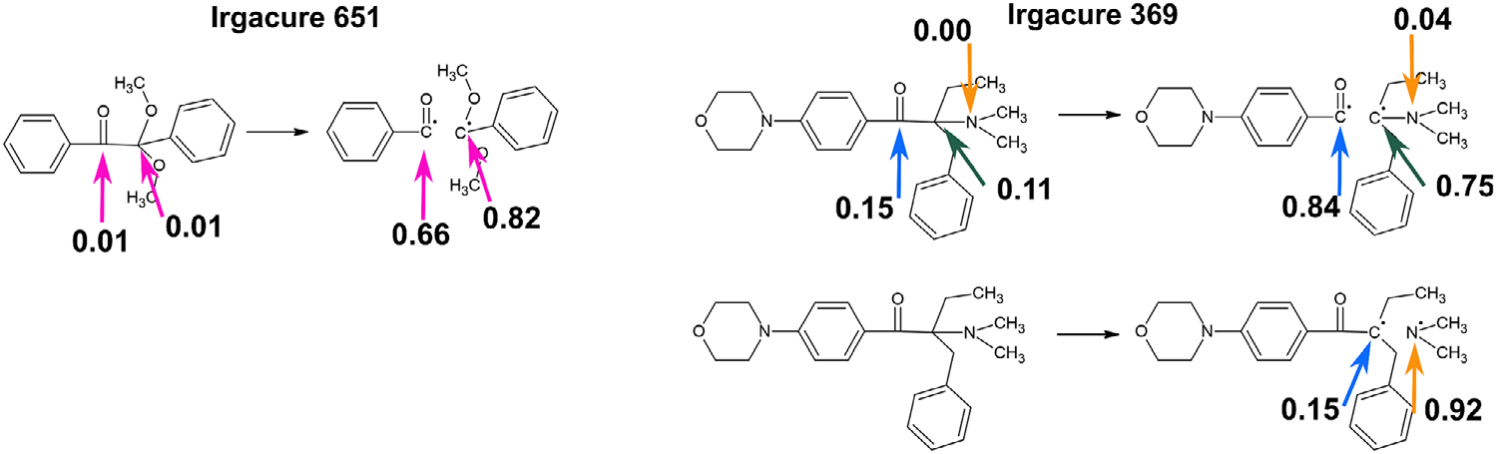
Fragmentation reaction mechanism with spin densities values on atoms for Irgacure 651 (α‐cleavage) and Irgacure 369 (α‐ and β‐cleavage). Results are reported in Table S26 (Supporting Information).

The calculated spin density values of the atoms involved in the cleavage reaction from the T_1_ state are reported in **Figure** **4** and Table S26 (Supporting Information). A higher spin density on certain atoms suggests a higher likelihood of radical character. Data obtained confirm that the homolytic cleavage of the C–C bonds (α‐cleavage of Irgacure 651 and Irgacure 369) and C–N (β‐cleavage of Irgacure 369) produces free radicals. Spin density increases, as expected, to higher values upon cleavage e.g., from 0.1 a.u. on the atom C12 and C13 to 0.66 and 0.82 a.u., respectively in the case of the α‐cleavage of Irgacure 651. The similar feature is observed for Irgacure 369, demonstrating the differences in spin densities depending on the cleavage type.

**Figure 5:**
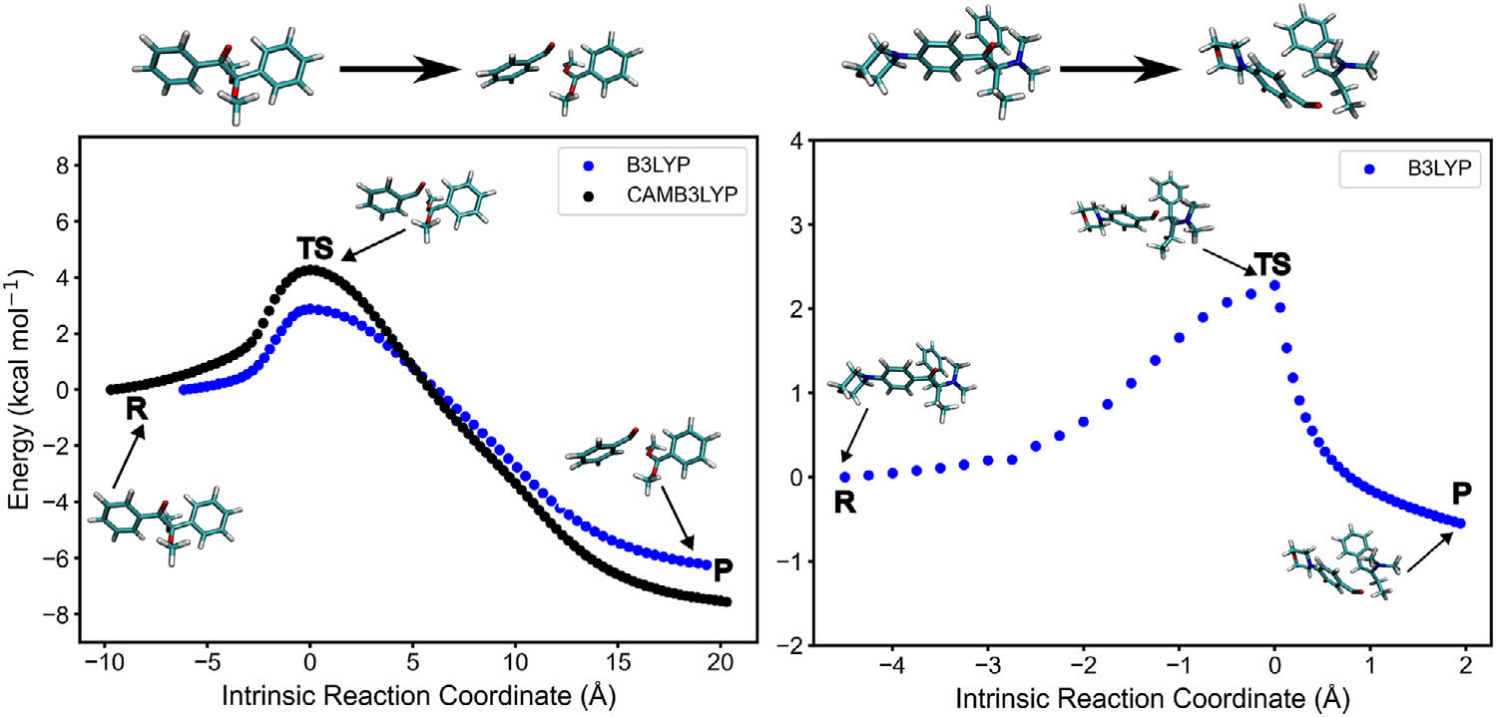
Norrish Type I fragmentation reaction of Irgacure 651 (α‐cleavage) computed with (U)CAM‐B3LYP‐D3(BJ)/def2TZVP (ACN) and (U)B3LYP‐D3(BJ)/def2TZVP in implicit ACN respectively (left) and Irgacure 369 (α‐cleavage) in (U)B3LYP‐D3(BJ)/def2TZVP in implicit ACN (right). The IRC profile was computed starting from the T_1_ optimized geometry (R) and moving through the TS, where the actual cleavage occurs, for reaching the formation of the products (P) upon relaxation and rearrangement.

To further investigate the α‐cleavage mechanism of both Norrish Type I PIs, a more detailed analysis involving the calculation of the energy barrier for the dissociation reaction from the T_1_ state was computed. For Irgacure 369, the CAM‐B3LYP‐D3(BJ)/def2‐TZVP level of theory was not able to converge the transition state, so results from B3LYP are presented (**Figure** **5**b). Considering that minor differences in BDEs and spin density values were observed across these two functionals we expect the results not to deviate significantly. For consistency, results for Irgacure 651 are reported using both functionals (Figure 5a).

The profile of the dissociation reaction involves first the initial elongation of the C–C bond starting from the optimized T_1_ geometry (R), subsequently the mentioned bond breaks with formation of a transition state (TS), and finally the two fragments rearrange. The lower energy of the product (P) relative to the reactant (R) in both PIs confirms the exothermic nature of the dissociation reaction. In the following, we will compare data obtained with B3LYP functional for both PIs. The first step involves the elongation of the respetive C–C bond (C12–C13 for Irgacure 651 and C25–C27 for Irgacure 369, see Figure S1, Supporting Information) by 0.45–0.48 Å, which was calculated as the difference between the bond lengths in the transition state and in the optimized T_1_ state. The activation barrier that must be overcome for the bond cleavage to occur is in the range 2.2–2.6 kcal mol^−1^, leading to reaction rate of 7.2 × 10^10^ s^−1^ and 2.5 × 10^11^ s^−1^ for Irgacure 651 and Irgacure 369, respectively. It significantly exceeds the non‐radiative T_1_‐S_0_ decay with the rate in the order of 10^5^ s^−1^ (see Table S22, Supporting Information). In agreement with the BDE energies explained above, this clearly indicates that for Norrish Type I PIs radicals are formed in the T_1_ state upon light absorption.

### 2.4 Functionality of BBK in FRP Initiation

#### 2.4.1 Triplet Absorption

**Figure 6:**
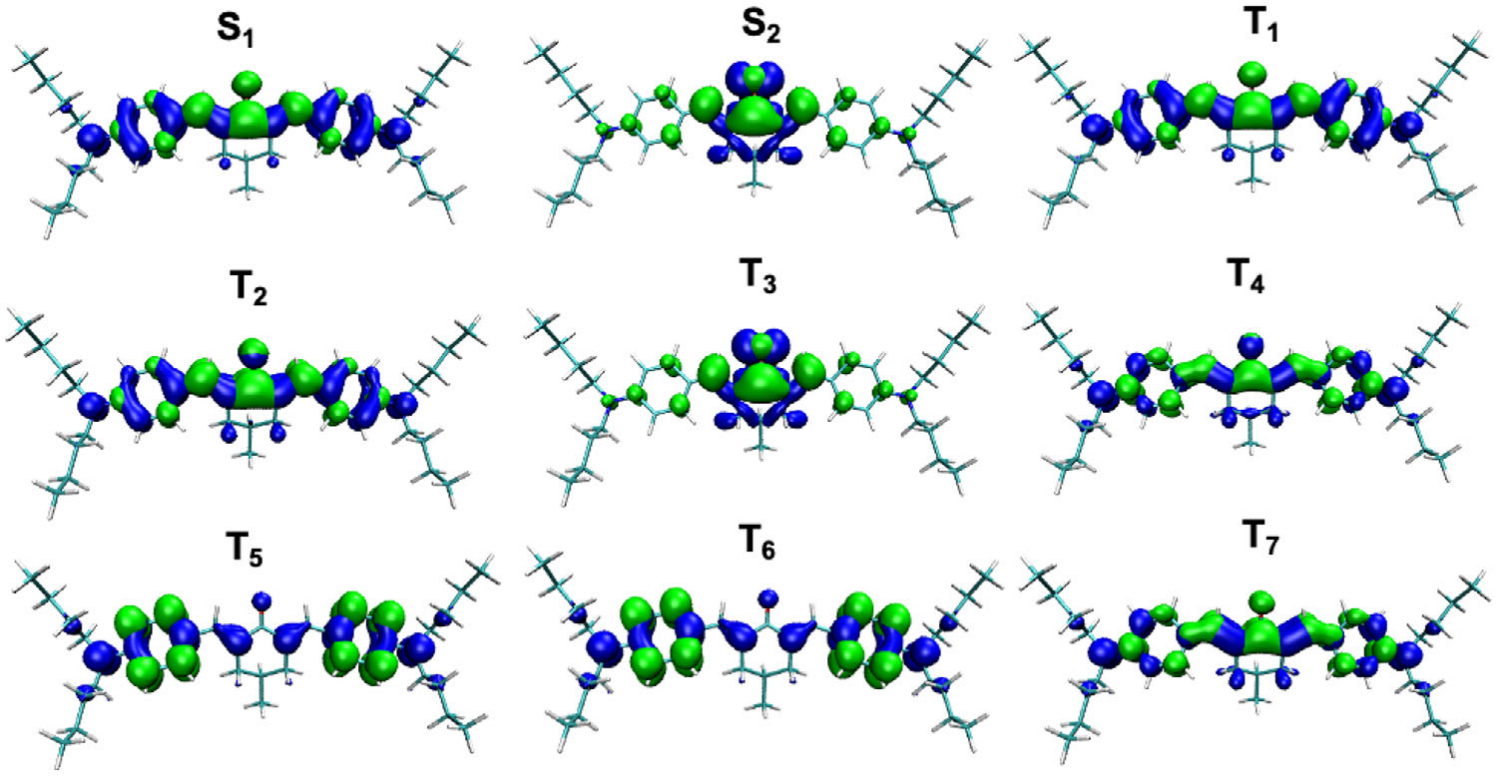
Visualization of electron donating (hole, in blue) and electron accepting (electron, in green) regions of BBK upon excitations from the ground state to singlet and triplet states in ACN. Electron‐hole analysis and the visualization of the respective contributions were performed based on data obtained using TDA‐CAM‐B3LYP‐D3(BJ)/def2‐TZVP level of theory. Singlet excited and triplet excited states are labeled with S and T, respectively. Isovalue of 0.001 a.u. was used for visualization.

In contrast to Norrish Type I PIs, Norrish Type II PIs as DETC^[^ ^19^ ^]^ and BBK^[^ ^26^ ^]^ are characterized by a distinct mechanism of photoactivation since the third‐order process is necessary to start the FRP in the absence of a co‐initiator.^[^ ^9^, ^19^, ^24^, ^25^, ^26^, ^33^, ^37^ ^]^ Therefore, it is assumed to involve a combination of a 2PA followed by 1PA in the triplet manifold (as depicted in Figure 1) with the radicals expected to be generated in high triplet states. We have discussed the suggested pathways for such a reactivity using the DETC initiator recently.^[^ ^19^ ^]^ Similarly to DETC, the charge density analysis of the singlet and triplet excitations of BBK, i.e., the change of the electron density during an excitation (see **Figure** **6**), shows a possible activation of carbonyl moiety upon the T_1_ excitation (atoms C1‐O12 see, areas in green). This may permit further hydrogen atom transfer (HAT) reactions with co‐initiators or the PETA monomers as known for this class of PIs. Applying the similar rule as for Norrish Type I, i.e., $E_{\text{bond}}^{\text{BDE}}$ < $E_{T_{1}}$, we see that the energy of T_1_ is lower than BDE of several bond breaking cases, i.e., 53.9 kcal mol^−1^ vs 68.3 ‐ 117.2 kcal mol^−1^ for the optimized T_1_ (see Table S27, Supporting Information). The only exception is the cleavage of C45‐N26 bond with a BDE of 51.3 kcal mol^−1^, which is however still in the range of uncertainty in DFT. This indicates a different reactivity of such PIs in the lowest triplet state and a lower probability of a bond cleavage.

**Figure 7:**
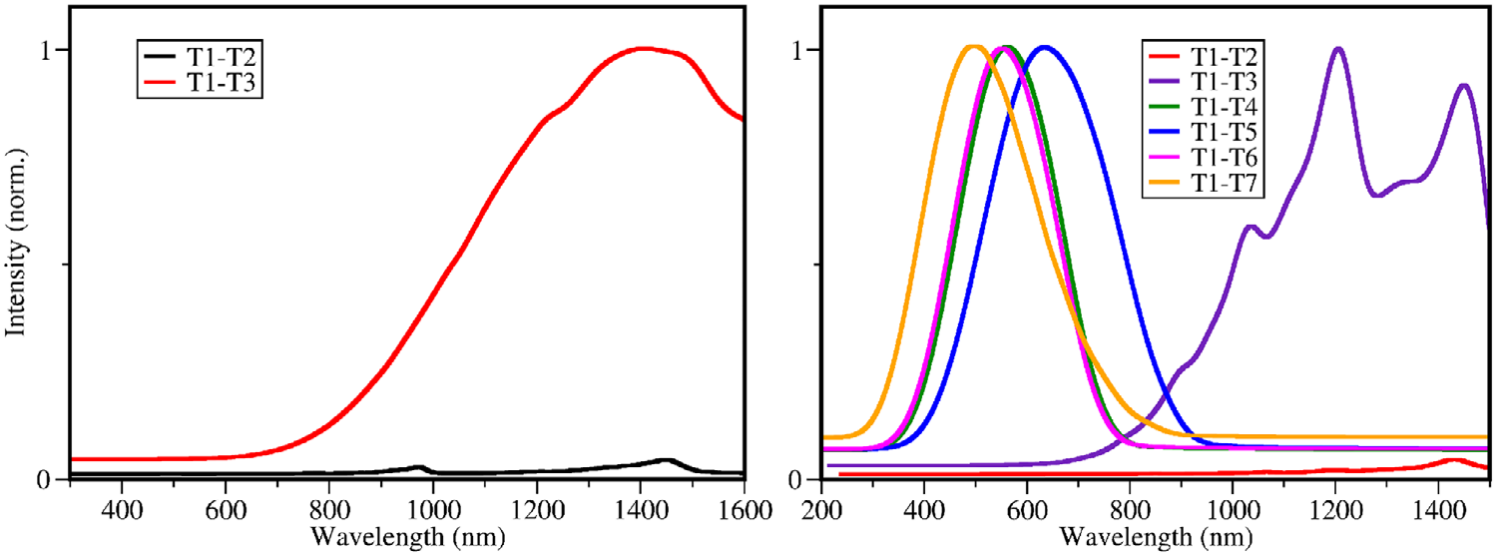
Vibrational triplet absorption spectra of BBK computed with CAM‐B3LYP (left) and B3LYP (right) as reported in Computational details.

In order to detect what triplet transition should then be the most probable upon the an absorption of the third photon, the electronic TA spectrum was computed (see Table S28, Supporting Information). Data shows absorption peak at 724 nm with an oscillator strength of 0.007 with CAM‐B3LYP. The comparison of TA differences between DETC and BBK is not yet reported experimentally; from the computed spectra with TD‐DFT it can only be seen that the TA of DETC is much stronger than for BBK: the oscillator strength of TA in DETC is 0.18 at 815.2 nm.^[^ ^19^ ^]^ It may explain the lower efficiency of the polymerization inhibition using BBK photoresists observed in experiments.^[^ ^37^ ^]^ In addition to the electronic spectrum, the vibrationally resolved TA spectra^[^ ^62^ ^]^ that consider the coupling of electronic and vibrational motion, related to the T_1_‐T_*n*_ transitions were computed (see **Figure** **7**). Data computed using CAM‐B3LYP shows a broadened absorption band at 800 nm and beyond for the T_1_‐T_3_ transition. It looks completely different than the absorption toward the T_1_‐T_2_ transition. Higher excited states could not be optimized, and therefore the respective TA spectra could not be computed. Data computed in B3LYP shows, similarly as for the CAM‐B3LYP case, that the transition from T_1_ would lead to the T_3_ state (or T_5_ state, considering the possible spectral shifts in the data calculated).

However, the formation of high triplet states may trigger deactivation of the BBK photoinitiator via the reversible intersystem crossing (RISC) back to the singlet manifold. Such a possibility was demonstrated experimentally^[^ ^37^ ^]^ and can indeed be observed by a rather high RISC rate between the T_3_ and S_1_ excited states. The rate calculated equals 2.1 × 10^9^ s^−1^ as reported in Table S23. Similar RISC rates were reported for DETC, i.e. rates of 5.4 × 10^9^ s^−1^ and 2.4 × 10^9^ s^−1^ for transitions from T_3_ and T_4_ to S_1_ excited state.^[^ ^19^ ^]^ The deactivation pathway prevents the formation of radicals necessary for FRP, introducing an additional level for the printing resolution control. As reported by Somers et al.,^[^ ^37^ ^]^ it may explain why the printing laser sometimes struggles to induce polymerization, requiring higher powers than expected.

#### 2.4.2 Formation of Radicals

**Figure 8:**
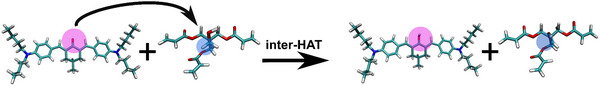
Radical formation mechanism characterized by intermolecular hydrogen atom transfer (inter‐HAT) between PETA and BBK. The schematic attack on position R_1_ of PETA is depicted. The definition of the possible active sites is explained in Figure S18 in Ref. [19].

A detailed analysis of the reactions leading to radical formation revealed that the monomer PETA plays a significant role in an inter‐HAT mechanism with the PI.^[^ ^19^ ^]^ Building on these findings, this work investigates a similar reaction between BBK and PETA. As reported for DETC, the mechanism involves the attack of the carbonyl bond of BBK on the C‐H bond of PETA, resulting in the formation of a ketyl radical on the PI and an alkyl radical on the monomer,^[^ ^19^ ^]^ as illustrated in **Figure** **8**.

Activation energy with the formation of the transition state in such a reaction was not computed due to the large number of atoms and the complexity of the reaction. Considering rather good agreement of reaction barriers and BDE in the case of Norrish Type I PIs (see Table 5 and Figure 5), the possibility of C–H bonds breaking of PETA were investigated. Based on the previously reported data,^[^ ^19^ ^]^ only the most probable radicals on PETA (R_1_ and R_2_) in the presence of BBK were studied. The results obtained are reported in Table 6. The Gibbs free energy of the reaction involving BBK in T_1_ with PETA (R_1_) is 2.3 kcal mol^−1^ (for comparison, it is 5.5 kcal mol^−1^ with DETC), while in S_0_, it is 47.3 kcal mol^−1^ (52.8 kcal mol^−1^ for DETC)(see Table 6). The values are rather similar and they indicate that the reaction can not happen if the PI is not excited, but the reaction with BBK in T_1_ is more achievable. However, when BBK is in higher triplet states (e.g., T_3_), a significantly higher degree of spontaneity is observed with Gibbs free energy in the range of –21.0 kcal mol^−1^. Since the trends for both BBK and DETC with this respect are rather similar, the hypothesized radical formation pathways are assumed to be similar. Some differences may be expected in the lowest triplet state, because the Gibbs free energy is on the level of the DFT energy uncertainties. Note that these values consider only to the thermodynamics of the reaction and the extension to the analysis of the kinetics should be performed to be able to determine with certainty the rate of the reaction.

**Table 6:** Gibbs free energies (in kcal mol^−1^) related to the Norrish Type II radical formation reactions through the inter‐HAT mechanism between PETA and BBK in the S_0_ state and triplet excited states. All calculations were performed at the (U)CAM‐B3LYP‐D3BJ/def2‐TZVP level of theory in implicit ACN. Data for the radicals of PETA in the reaction with DETC reported previously^[^ ^19^ ^]^ are given for comparison.

Radical of PETA	S_0_	T_1_	T_2_	T_3_
R_1_ ‐ BBK	47.25	2.34	−9.33	−21.02
R_2_ ‐ BBK	46.96	2.05	−9.63	−21.31
R_1_ ‐ DETC	52.77	5.47		−20.14
R_2_ ‐ DETC	52.47	5.18		−20.43

Beyond the reaction between the BBK and the PETA monomer, the analysis of several other possible radical formation mechanisms, such as photoactivated H‐abstraction and photolysis, was conducted on the basis of the calculated for DETC.^[^ ^19^ ^]^ These reactions were evaluated through Gibbs free energy. The photoactivated H‐abstraction refers to cleavage of C–H bonds of BBK with the abstraction of H‐atom, as reported in Ref. [63]. This results are given in Table S30 and Figure S14 (Supporting Information). Data shows that the H‐atom abstraction from BBK is characterized by rather high Gibbs free energies with the lowest values of around 20 kcal mol^−1^ (CAM‐B3LYP) in T_3_ from the CH_2_ group near the N atom in accordance with the presence of heteroatoms with higher electronegativity than C, which facilitate the cleavage.^[^ ^63^ ^]^ The positive Gibbs free energies related to all studied C‐H cleavages in different excited states show that this process should not occur during FRP of PETA.

**Figure 9:**
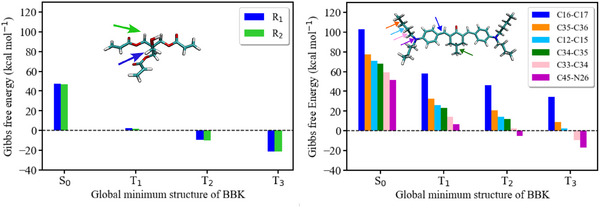
Gibbs free energy for the reaction between BBK and PETA (up) and photolysis of different bonds of BBK (down) calculated using (U)CAM‐B3LYP‐D3(BJ)/def2‐TZVP approach in implicit ACN. The bonds labeled are depicted in Figure S2 (Supporting Information).

In the case of photolysis, the Gibbs free energy associated to the cleavage of the C33–C34 and C45–N26 bonds of BBK (see **Figure** **9**; Table S31, Supporting Information) is –9.3 kcal mol^−1^ and –17.0 kcal mol^−1^ from T_3_, respectively. These values indicate that the process is energetically similar to the reaction with PETA from the same state (the Gibbs free energy of –21.0 kcal mol^−1^, see Table 6 and Figure 9). It resembles the observations obtained for DETC, however, BBK possesses two tertiary amine groups, thus, more active species may be formed as a result of such a reaction. Since the quantum yield of active radical formation is essential for efficient photoinitiators, it may be one of the reasons for the better performance of BBK in 3D printing in comparison to DETC^[^ ^41^, ^42^ ^]^ in addition to slightly lower Gibbs free energies for the HAT with PETA. The differences in fluorescence quantum yield of these photoinitiators considering PETA solutions may also play a role, thus, further studies in this direction would be beneficial.

## 3 Conclusion 

In this study, we conducted an extensive investigation of various PIs, focusing on the complex reaction processes that lead to the formation of radicals, which subsequently activate FRP during 3D laser printing. This marks the first detailed comparison between Norrish Type I PIs, which generate radicals through the absorption of two photons and subsequent bond cleavage, and a special category of Norrish Type II PIs that activate polymerization via a unique third‐order process.

To characterize the multiphoton absorption, as well as decay pathways of the PIs, we analyzed their absorption properties, charge transfer characteristics and rates of radiative and non‐radiative decays. As a result we could show that Irgacure 651, Irgacure 369, and BBK exhibited 2PA in the 600–800 nm range, however, the calculated 2PA cross‐section for BBK is higher. Upon optimization of several excited states (singlets and triplets) and computing the related photophysical rates, we found that Irgacure 651 and Irgacure 369 populate the T_1_ state after 2PA and further ISC that occurs with the rate of 4.5 × 10^7^ s^−1^ and 7.9 × 10^7^ s^−1^, respectively. After ISC, α‐cleavage of the bonds C12‐C13 (Irgacure 651) and C25–C27 (Irgacure 369) takes place with rates of 7.2 × 10^10^ s^−1^ and 2.5 × 10^11^ s^−1^, respectively, producing the benzoyl and alkyl radicals for the former, α‐alkyl and α‐alkylamino radicals for the latter. They initiate polymerization in 3D laser printing. This is further supported by the computation of BDE, reaction energy barriers and spin densities. In the case of BBK, ISC is slower and the T_1_ state formed has a relatively long lifetime (ranging from 3 to 11 µs depending on the DFT functional), allowing to absorb the third photon. In comparison to the Norrish Type I PI, the bond cleavage is less probable to occur in this state.

The triplet absorption of BBK promotes the system to even higher triplet states, most probably T_3_, as further confirmed by the analysis of the vibrational absorption spectra. Since polymerization happens without any co‐initiator (as typically used for Norrish Type II PIs), different FRP pathways than known for Norrish Type I PI are triggered. In addition, the BBK deactivation due high RISC rates from the high triplet states may occur, permitting higher 3D laser printing resolution. From the analysis of Gibbs free energies, we observe that the most probable is the intermolecular HAT between the monomer PETA and BBK, as it was for explained for DETC, permitting the formation of a ketyl radical on the PI and an alkyl radical on the monomer. The values for the reaction with BBK in T_1_ indicate non‐spontaneity, however, values are lower than for DETC and are on the order of possible DFT uncertainties. In addition, another possible radical formation reaction, according to the Gibbs free energy, is the photolysis of carbon‐carbon (C33–C34) and carbon‐nitrogen (C45–N26) bonds, although still less spontaneous than the inter‐HAT with the monomer.

The findings on BBK show marked similarities with DETC, reinforcing the fact that both of these PIs are highly efficient for 3D printing applications and that both operate through a unique third‐order process. However, there is a delicate difference in Gibbs free energies for the HAT with PETA, and BBK possesses two tertiary amino groups, i.e. more radical species if the C45‐N26 bond breaks may be formed. Thus, BBK may have a higher quantum yield of active radical formation, which may be further modulated by the differences in fluorescence quantum yield. Experimental investigations of these processes would be beneficial. Prominent differences observed for these PIs are in the charge transfer character of the S_1_ excited state (BBK has less CT) and absorption characteristics: the 2PA cross‐sections may be higher for BBK, while the TA absorption is much stronger for DETC. However, the studies reported here were conducted in implicit solvent and without the impact of diffusion during 3D printing, leaving an open space for further studies in the field.

## 4 Computational Details

Gaussian16 Rev.C.01^[^ ^64^ ^]^ was used to optimize all geometries of ground states and excited states employing a default ultra‐fine grid for numerical integrations and an energy convergence criterion of 10^−8^ Hartree. The solvent ACN was treated implicitly with polarizable continuum implicit solvent model (PCM). A non‐equilibrium solvation method was used for single‐point calculations, while for the optimization of excited states the equilibrium solvation was used, as default in Gaussian16 Rev.C.01. The optimized geometries were confirmed through vibrational and molecular orbitals analysis. The excited states of Norrish Type I PIs were optimized with TD‐CAM‐B3LYP‐D3(BJ)/def2‐TZVP in ACN and the gas‐phase (due to convergence problems of the correlation function when employing the solvent). The optimization of the excited states of BBK was performed with both TDA‐CAM‐B3LYP‐D3(BJ)/def2‐TZVP and TDA‐B3LYP‐D3(BJ)/def2‐TZVP in ACN.

The 1PA and 2PA^[^ ^65^, ^66^, ^67^ ^]^ spectra of the PIs investigated were performed on the optimized ground state geometry with Dalton 2020.0^[^ ^68^ ^]^ employing the PCM model^[^ ^69^, ^70^ ^]^ for ACN and using TD‐CAM‐B3LYP^[^ ^71^ ^]^/def2‐TZVP^[^ ^48^, ^72^, ^73^ ^]^ with the addition of TDA^[^ ^74^ ^]^ for BBK. For the letter, the TDA‐B3LYP^[^ ^75^ ^]^/def2‐TZVP level of theory was also used for comparison with the most of data available in Supporting Information. Three‐photon absorption (3PA) spectra^[^ ^66^, ^73^, ^76^ ^]^ of BBK were computed in Dalton 2020.0 with TDA‐CAM‐B3LYP/def2‐SVP and TDA‐B3LYP/def2‐SVP in the gas phase. The def2‐SVP basis set was used to reach convergence. 2PA and 3PA absorption spectra were calculated simulating the experimental setup with a double laser beam, linearly polarized light, and parallel polarization.^[^ ^65^ ^]^ Transition moments were defined for two and three photons of the same frequency, respectively. All spectra were plotted with half‐width‐of‐half‐maximum (HWHM) of 0.1 eV and Lorentzian‐type broadening function. For further details on the theory please refer to our previous work.^[^ ^19^ ^]^

The TA spectra for BBK were calculated in ACN using the unrestricted approach, i.e., TD‐(U)CAM‐B3LYP‐D3(BJ)^[^ ^77^ ^]^/def2‐TZVP and TDA‐(U)CAM‐B3LYP‐D3(BJ)/def2‐TZVP approach, respectively employing the optimized geometry of T_1_ as the starting point. Spectra were performed with Gaussian16 Rev.C.01. The vibronically resolved TA spectra of BBK were computed employing the Frank‐Condon (FC)^[^ ^62^, ^78^, ^79^ ^]^ approximation within the linear coupling model (LCM),^[^ ^80^, ^81^ ^]^ not considering Duschinsky rotation.^[^ ^82^ ^]^ Spectra were generated at 100K with a Lorentzian broadening, HWHM of 80 cm^−1^ (0.01 eV) and convergence factor of 1.0x10^−4^ a.u using Dynavib.^[^ ^83^, ^84^ ^]^ The previously optimized geometries in Gaussian 16 Rev.C.01 were used as input structures.

MOMAP (Molecular Materials Property Prediction Package) 2022A (version 2.3.3)^[^ ^85^, ^86^, ^87^, ^88^, ^89^, ^90^ ^]^ based on thermal vibration correlation function (TCVF) was used to computed the radiative and non‐radiative rates reported in this work using as input the optimized geometries in Gaussian16 Rev.C.01 (including zero point vertical energy). The interval of the correlation function was set to 1000 fs and the integration timestep of the correlation function (dt) to 0.01 fs neglecting the Duschinsky rotation (except for the computation of ISC and RISC of Irgacure 651). Adiabatic Hessian (AH) model of the PES was selected. Non‐adiabatic coupling elements (NACME)^[^ ^91^, ^92^, ^93^ ^]^ were calculated in Gaussian16 Rev.C.01, while SOC^[^ ^94^ ^]^ with ADF 2020.1,^[^ ^95^ ^]^ utilizing the respective optimized structures. SOC was calculated in ACN with TZP basis set employing CAM‐B3LYP (and B3LYP for BBK) at the scalar level within ZORA formalism,^[^ ^82^, ^96^, ^97^ ^]^ adding non‐equilibrium properties. SOC to compute the S_0_‐T_1_ rate was calculated in ORCA Version 4.2.1^[^ ^98^, ^99^ ^]^ employing the CPCM^[^ ^100^ ^]^ model for ACN using quasi‐degenerate perturbation theory and CAM‐B3LYP (and B3LYP for BBK). In the case of BBK TDA was included for the computation of SOC.

The computation of the bond cleavage (i.e., C–C, C–H, C–N) of the PIs investigated in this work was performed with both BDE^[^ ^101^ ^]^ and Gibbs free energy with unrestricted scheme i.e.,(U)CAM‐B3LYP‐D3(BJ)/def2‐TZVP and (U)B3LYP‐D3(BJ)/def2‐TZVP in the similar way as in Ref. [19]. The dissociation reaction from the T_1_ state was computed using IRC,^[^ ^102^ ^]^ as implemented in Gaussian16 Rev.C.01, considering the forward and reverse direction of the reaction starting from the optimized first triplet excited state. The transition state geometry was verified by vibrational analysis, and the final activation energy barrier was calculated with the Eyring^[^ ^103^ ^]^ equation. Temperature of 298 K and pressure of 1 atm were considered with (U)CAM‐B3LYP‐D3(BJ)/def2‐TZVP and (U)B3LYP‐D3(BJ)/def2‐TZVP approach.

## Conflict of Interest

The authors declare no conflict of interest.

## Supporting information

Supporting Information

## Data Availability

The data that support the findings of this study are available from the corresponding author upon reasonable request.
