# Supplementary material for: Photoreactivity of Norrish Type Photoinitiators for 3D Laser Printing via First Principles Calculations
Source: Macromol Rapid Commun. 2025 May 15;46(23):2500231. doi: 10.1002/marc.202500231 (PMC12687724; doi:10.1002/marc.202500231)
Supplement: Supplementary file 1 — Supporting Information [file MARC-46-2500231-s001.pdf]

Supplementary Information

Photoreactivity of Norrish Type Photoinitiators for 3D Laser Printing via First Principles Calculations

*Anna Mauri Pascal Kiefer Wolfgang Wenzel Mariana Kozlowska**

Dr. Anna Mauri, Prof. Dr. Wolfgang Wenzel, Dr. Mariana Kozlowska
Address: Institute of Nanotechnology (INT), Karlsruhe Institute of Technology (KIT), Kaiserstraße 12, 76131 Karlsruhe, Germany
Email: mariana.kozlowska@kit.edu

Dr. Pascal Kiefer
Address: Institute of Applied Physics (APH), Karlsruhe Institute of Technology (KIT), Kaiserstraße 12, 76131 Karlsruhe, Germany

1 Molecules studied

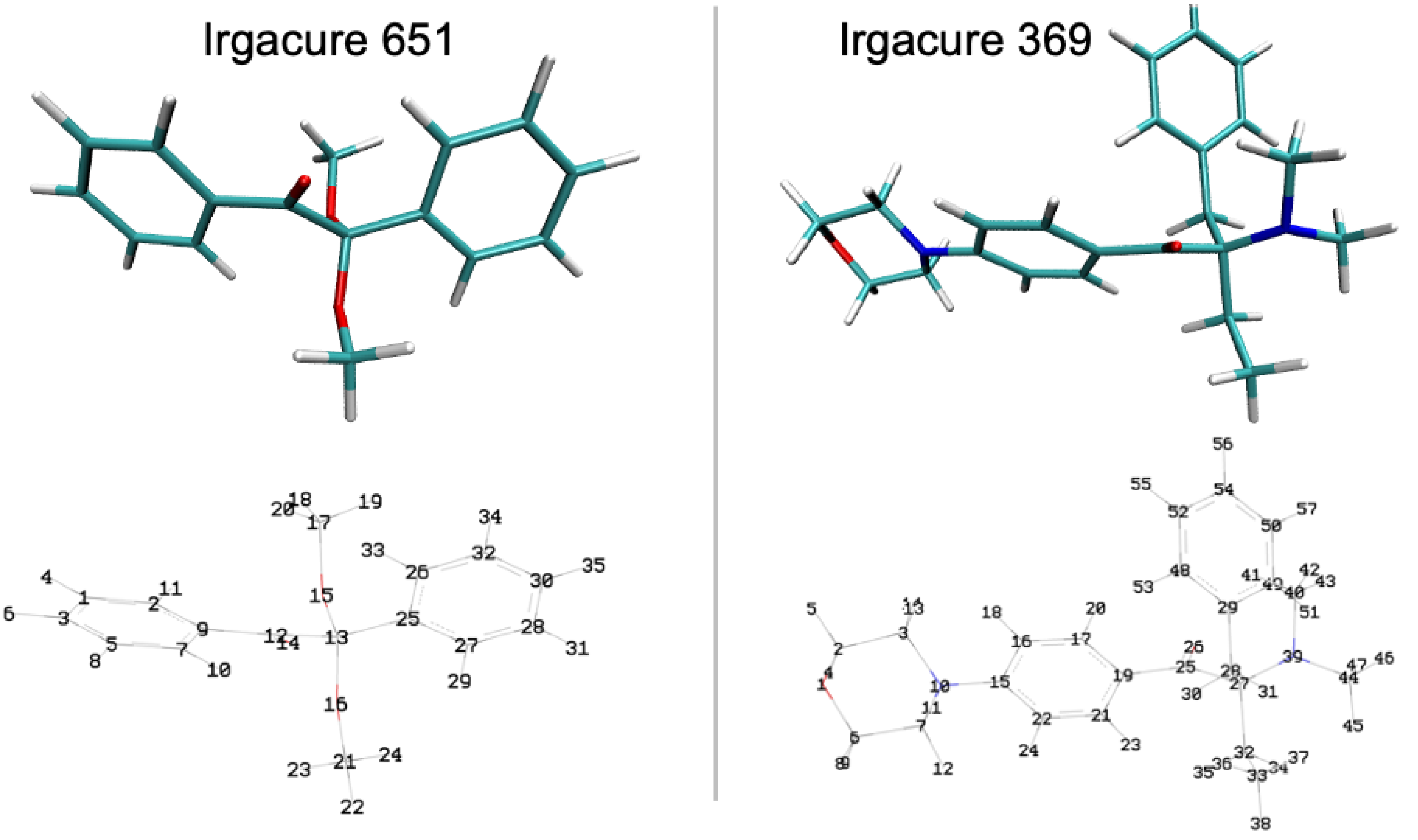

Figure S1: Graphical representation of Irgacure 369 and Irgacure 651 with the respective atom numbering.

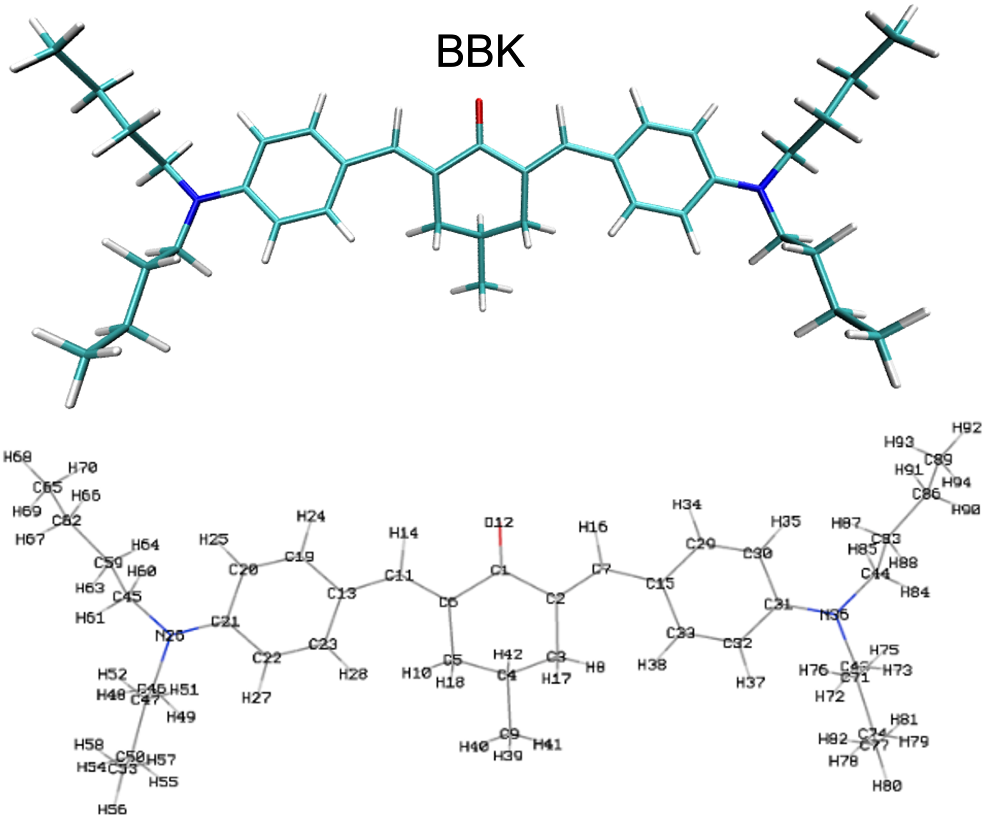

Figure S2: Graphical representation of BBK with the respective atom numbering.

2 One-photon absorption and emission properties

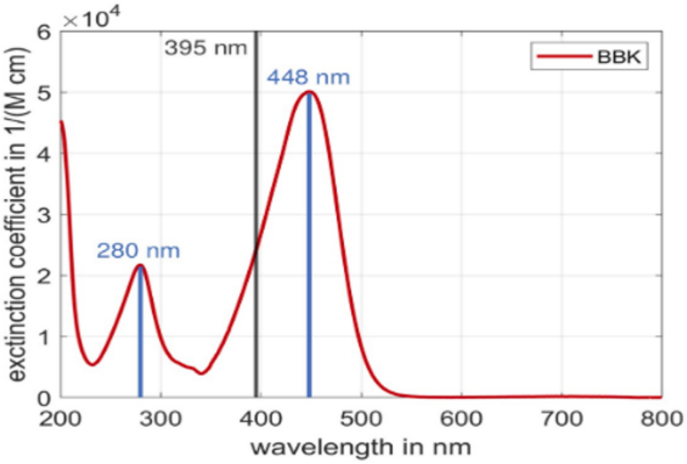

Figure S3: Experimental absorption spectrum of BBK in ACN.

Table S1: Vertical excitation energies (energy in eV and wavelength in nm) for singlet excitations of Irgacure 651 in ACN calculated using TD-CAM-B3LYP-D3(BJ)/def2-TZVP, starting from the optimized ground state geometry (S_0). Experimental absorption maximum is in the range of 250-341 nm[1, 2].

Excitation	Energy	Wavelength	Osc. str.
1	3.78	328.45	0.0022
2	4.94	250.76	0.0267
3	5.14	241.09	0.0378
4	5.46	226.92	0.0067
5	5.69	218.03	0.0020
6	5.76	215.32	0.0022
7	6.05	204.87	0.0019
8	6.09	203.51	0.0549
9	6.22	199.44	0.0076
10	6.29	197.00	0.0007

Table S2: Vertical excitation energies (energy in eV and wavelength in nm) for singlet excitations of Irgacure 369 in ACN calculated using TD-CAM-B3LYP-D3(BJ)/def2-TZVP, starting from the optimized ground state geometry (S_0). Experimental absorption range is between 200 and 425 nm depending on the concentration[2, 3].

Excited State	Energy	Wavelength	Osc. str.
1	4.09	303.50	0.0278
2	4.22	294.04	0.0895
3	4.41	281.35	0.5447
4	4.75	261.15	0.0210
5	5.31	233.61	0.0099
6	5.53	224.25	0.0487
7	5.70	217.36	0.1058
8	5.89	210.60	0.0102
9	5.98	207.37	0.0285
10	6.13	202.12	0.0217

Table S3: Vertical excitation energies (energy in eV and wavelength in nm) for the first ten singlet excitations of BBK in ACN calculated using TDA-B3LYP-D3(BJ)/def2-TZVP and TDA-CAM-B3LYP-D3(BJ)/def2-TZVP method, starting from the respective optimized ground state geometry (S_0). The oscillator strength (Osc.str.) values for triplet excitations are zero and therefore not reported. Experimental absorption peak in ACN and toluene is at 448 nm and 435 nm, respectively[4].

		**B3LYP**			**CAM-B3LYP**	
State	Energy	Wavelength	Osc. str.	Energy	Wavelength	Osc. str.
1	2.65	467.67	1.9698	3.28	377.62	2.1643
2	3.09	401.77	0.0250	3.63	341.75	0.0370
3	3.25	381.45	0.0057	3.83	323.62	0.0364
4	4.15	298.97	0.0162	4.56	271.73	0.0127
5	4.15	298.86	0.0525	4.57	271.57	0.0701
6	4.27	290.34	0.7635	5.09	243.41	0.0086
7	4.29	289.14	0.0179	5.26	235.75	0.3261
8	4.45	278.38	0.0019	5.40	229.46	0.0069
9	4.47	277.22	0.0051	5.45	227.43	0.1916
10	4.51	274.87	0.0010	5.58	222.32	0.0140

Our previous work[5] has highlighted the crucial impact of solvent dynamical changes on the selection of the DFT functional, a key aspect in DFT studies. Common practice, as reported extensively in the literature, limits functional choice to the analysis of vertical excitation energies from optimized ground-state geometries and/or zero-point energy (ν_{00} energy), comparing them to experimental data. However, this approach has limitations, as DFT functionals that match experimental absorption spectra and ν_{00} energy may still produce inaccurate emission properties, especially when non-negligible charge transfer (CT) effects are present. This underscores the need to go beyond traditional comparisons of vertical excitation and E_{00} energies with experiments, integrating corrected linear response (cLR) methods to improve reliability and provide a more thorough assessment of functional accuracy. In this study, we determined the most suitable functional by comprehensively analyzing the absorption and emission characteristics of the PIs, using optimized geometries for both the ground state and first singlet excited state in solution, with the polarizable continuum model (PCM) for ACN. Specifically, we evaluated the absorption and emission properties using both linear response (LR) and corrected linear response (cLR) approaches to ensure robust results.

Table S4: Vertical excitation energies of Irgacure 651 and Irgacure 369 in implicit ACN employing linear response (LR) and corrected LR (cLR) approach. Experimentally estimated absorption maximum at ACN is 341.55 nm (3.63 eV) and 320.37 (3.87 eV) for Irgacure 651 and Irgacure 369, respectively. Data were computed starting from the optimized geometry with the respective functional.

	**LR**		**cLR**	
	eV	nm	eV	nm
**Irgacure 651**				
B3LYP	3.47	357.30	3.44	360.42
CAM-B3LYP	3.78	328.00	3.77	328.87
**Irgacure 369**				
B3LYP	3.22	385.04	3.00	413.28
CAM-B3LYP	4.09	303.14	4.07	304.63

Table S4 shows that data computed with LR and cLR approach in CAM-B3LYP are consistent and deviates by 0.14-0.15 eV for Irgacure 651 and 0.20-0.22 eV for Irgacure 369 from experimental data. On the contrary, data computed with B3LYP functional, especially in the case of Irgacure 369 show a much larger deviation from experiment i.e. 0.65-0.87 eV.

Table S5: Adiabatic and E_{00} energy values for Irgacure 651 and Irgacure 369 computed with B3LYP and CAM-B3LYP with linear response (LR) and corrected LR (cLR) approach including zero-point energy correction (ZPVE).

	**B3LYP**		**CAM-B3LYP**	
	eV	nm	eV	nm
	**Irgacure 651**			
LR $E_{adiabatic}$	3.13	396.12	3.53	351.23
LR E_{0-0}	3.02	410.54	3.43	361.47
cLR $E_{adiabatic}$	2.96	418.87	3.46	358.34
cLR E_{0-0}	2.85	435.03	3.36	369.00
	**Irgacure 369**			
LR $E_{adiabatic}$	2.51	493.96	3.31	354.57
LR E_{0-0}	2.44	508.13	3.27	379.16
cLR $E_{adiabatic}$	1.92	645.75	3.01	411.91
cLR E_{0-0}	1.86	666.58	2.97	417.46

In accordance to what was outlined previously, Table S5 demonstrates that using CAM-B3LYP cLR to account for solvent dynamical changes yields E_{0-0} energy values for both Norrish type I PIs—3.36 eV and 2.97 eV—that closely match experimental data. In contrast, the E_{0-0} energy of 1.86 eV calculated for Irgacure 369 with B3LYP is significantly lower than experimental values. Further supporting this observation, Table S6 shows that B3LYP-calculated emission energies for both PIs are considerably underestimated, with values of 2.25–2.34 eV for Irgacure 651 and 0.96–1.27 eV for Irgacure 369, whereas CAM-B3LYP data remain consistent with experimental values.

This discrepancy can be attributed to the relaxation behavior of the S_1 state in ACN relative to S_0, often indicative of significant charge-transfer (CT) character, which B3LYP fails to capture due to its incorrect asymptotic behavior in the exchange-correlation potential [6, 7]. Solvent-induced effects on molecular orbitals may also contribute to this deviation. For these reasons, the CAM-B3LYP functional is chosen to accurately describe the properties of both Irgacure 651 and Irgacure 369.

Table S6: Comparison of the emission energy (fluorescence) of Irgacure 651 and Irgacure 369 computed with B3LYP and CAM-B3LYP using several approaches. The S_0 and S_1 geometry were optimized firstly with DFT and TD-DFT using the respective functional. The equilibrium corrected linear response (cLR eq.), and non-equilibrium cLR (cLR noneq.) were computed as a single point on the S_1 minimum geometry obtained with TDA-DFT with both functionals.

	TD-DFT		cLR noneq*		cLR eq**	
	nm	ev	nm	eV	nm	eV
			Irgacure 651			
B3LYP	410.54	3.02	529.85	2.34	551.04	2.25
CAM-B3LYP	361.47	3.43	387.45	3.20	392.36	3.16
			Irgacure 369			
B3LYP	508.13	2.44	976.25	1.27	1291.50	0.96
CAM-B3LYP	379.16	3.27	712.55	1.74	779.77	1.59

*Emission corrected linear-response approach (non-equilibrium solvation based. **Emission corrected linear-response approach (equilibrium solvation based: the corrected linear-response equilibrium solvation of the excited state S_1 at its equilibrium geometry).

Similar analysis was conducted for BBK. Data reported in Table S7 shows that B3LYP and CAM-B3LYP functional shows similar results, the latter being closer to experiment with a deviation of 0.05 eV. When considering the adiabatic E_{0-0} energy again both functionals show reasonable values e.g. 3.13 eV and 2.63 eV in cLR. The deviation to experiment is larger with B3LYP (0.37 eV) that with CAM-B3LYP (0.13 eV) but still without any strong change in the values (see Table S8). Similarly, for the fluorescence energies the values are 2.51 eV and 2.87 eV in CAM-B3LYP and B3LYP, respectively (see Table S9), maintaining the mentioned consistency. This observation prompted the adoption of both B3LYP and CAM-B3LYP functional for calculating BBK, due to the problems in the optimization of excited states using the latter, as described further in Section 6.

Table S7: Vertical excitation energies of BBK in implicit ACN employing linear response (LR) and corrected LR (cLR). Experimentally estimated absorption maximum at ACN is 448 nm (2.76 eV). Data were computed starting from the optimized geometry with the respective DFT functional.

	**LR**		**cLR**	
	eV	nm	eV	nm
B3LYP	3.28	378.00	3.32	373.45
CAM-B3LYP	2.70	459.80	2.71	457.51

Table S8: Adiabatic and E_{0-0} energy values for BBK computed with B3LYP and CAM-B3LYP with linear response (LR) and corrected LR (cLR) approach including zero-point energy correction (ZPVE).

	**B3LYP**		**CAM-B3LYP**	
	eV	nm	eV	nm
LR $E_{adiabatic}$	2.43	510.22	2.93	422.64
LR E_{0-0}	2.35	527.59	2.87	432.00
cLR $E_{adiabatic}$	3.13	396.12	2.63	471.42
cLR E_{0-0}	3.07	403.86	2.55	486.21

Table S9: Comparison of the emission energy (fluorescence) of BBK computed with B3LYP and CAM-B3LYP using several approaches. The S_0 and S_1 geometry were optimized firstly with DFT and TD-DFT using the respective DFT functional. The equilibrium corrected linear response (cLR eq. and non-equilibrium cLR (cLR noneq.)), were computed as a single point on the S_1 minimum geometry obtained with TDA-DFT with both functionals.

	TD-DFT		cLR noneq*		cLR eq**	
	nm	eV	nm	eV	nm	eV
B3LYP	529.85	2.34	426.07	2.91	432.00	2.87
CAM-B3LYP	457.51	2.71	486.21	2.55	493.96	2.51

*Emission corrected linear-response approach (non-equilibrium solvation based). **Emission corrected linear-response approach (equilibrium solvation based: the corrected linear-response equilibrium solvation of the excited state S_1 at its equilibrium geometry).

Table S10: Fluorescence peaks of Irgacure 651 and Irgacure 369 in ACN (in nm and eV) and oscillator strength (f) computed with CAM-B3LYP-D3-(BJ)/def2-TZVP in implicit ACN. Emission spectra in the experiment are not available and therefore not reported in the Table.

	**Irgacure 651**	**Irgacure 369**
Emission (nm)	379.65	606.99
Emission (eV)	3.27	2.04
Oscillator strength (f)	0.0014	0.0014

The computed emission peaks in Table S10 at 379.65 nm and 606.99 nm shows a very large Stoke shift[8, 9] for Irgacure 369 (∼300 nm) in comparison to Irgacure 651 (∼50 nm), which might be attributed to a significant relaxation occurring after absorption, which is explained below.

3 Charge-transfer (CT) properties

Figure S4 shows the similarities and differences of the optimized first singlet state S1 and the ground state. In the case of Irgacure 651, almost no differences can be detected (Figure S4**a**), while, in the case of Irgacure 369, differences can be seen between the geometries optimized in solvent and in the gas phase (Figure S4**c**). While the gas-phase S_1 geometry closely mirrors the ground state structure, the S_1 geometry obtained in ACN shows substantial deviations (Figure S4**b**). In the latter, a pronounced relaxation is observed compared to the S_0 geometry, resulting in a significant twist of the side group to -6.62° with respect to the (C16C15N10C3) dihedral angle (Figure S4**d**). In contrast, the twist in the S_0 geometry is 32.10°. Conversely, the S_1 geometry optimized in the gas phase exhibits a twist of 57.55°

(47.3° in S_0), as shown in Figure S4**c**. The substantial relaxation seen in the S_1 ACN-optimized geometry, similar to our previous findings on DETC[5], can be attributed to the combination of the CT character of the S_1 state and solvent-induced polarization effects, explaining the large Stokes shift for this PI. The lack of experimental emission data prevents further comparison with the theoretical calculations.

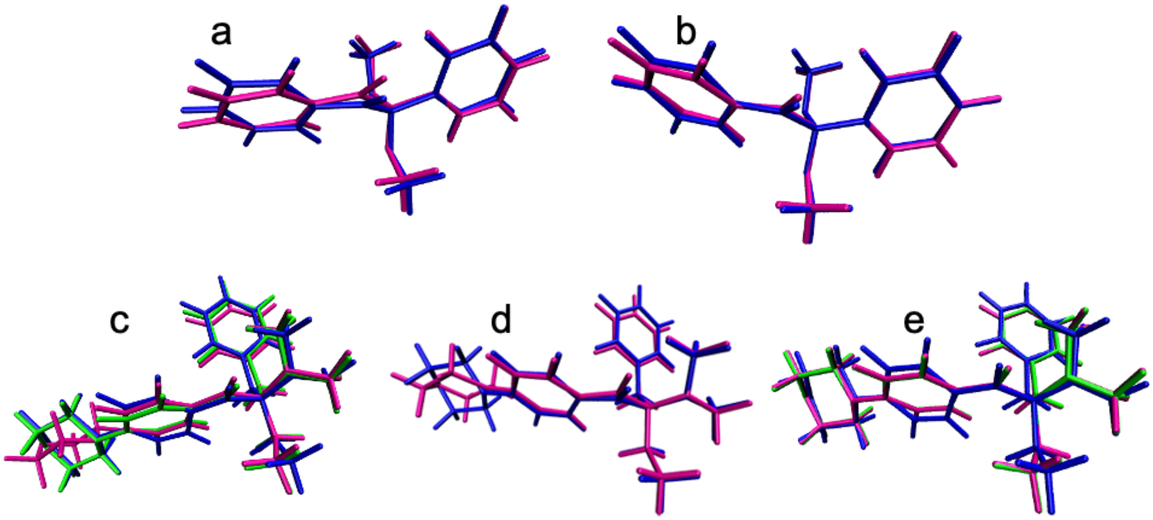

Figure S4: Graphical comparison of S_0, S_1 and T_1 optimized geometries of Irgacure 651 (**a**,**b**) and Irgacure 369 (**c**,**d**) with CAM-B3LYP: **a** ground state in ACN (in blue) to S_1 in ACN (in magenta), **b** ground state in ACN (in blue) to T_1 in ACN (in magenta), **c** ground state in ACN (in blue) to S_1 in ACN (in magenta) and to S_1 in the gas phase (in green), **d** ground state in ACN (in blue) to T_1 in ACN (in magenta), **e** ground state in gas (blue) with S_1 (magenta) and T_1 (green) in the gas phase.

Table S11: Transition molecular orbitals (MOs) and contributions for Irgacure 651 in ACN with CAM-B3LYP-D3(BJ)/def2-TZVP level of theory.

**Transition**	**Molecular Orbitals**	**Contribution** (%)
$S_0 \to S_1$	HOMO → LUMO	51.0
$S_0 \to T_1$	HOMO → LUMO	46.8

Table S12: Transition molecular orbitals (MOs) and contributions for Irgacure 369 in ACN with CAM-B3LYP-D3(BJ)/def2-TZVP level of theory.

**Transition**	**Molecular Orbitals**	**Contribution (%)**
$S_0 \to S_1$	HOMO -1 → LUMO	11.4
	HOMO -2 → LUMO	7.6
	HOMO -3 → LUMO	20.8
	HOMO -4 → LUMO	34.1
	HOMO -5 → LUMO	12.3
$S_0 \to T_1$	HOMO → LUMO	38.0
	HOMO -1 → LUMO	38.7

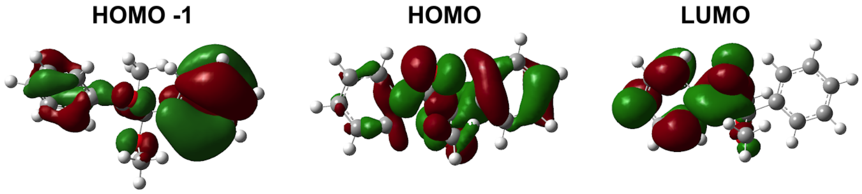

Figure S5: Visualization of molecular orbitals (MOs) of Irgacure 651 in ACN obtained using CAM-B3LYP-D3(BJ)/def2-TZVP level of theory. HOMO and LUMO correspond to the highest occupied molecular orbital and lowest unoccupied molecular orbital, respectively. Isovalue of 0.002 a.u. was used for visualization.

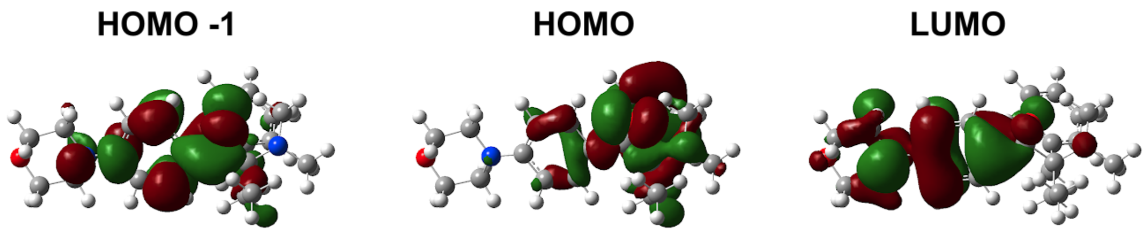

Figure S6: Visualization of molecular orbitals of Irgacure 369 in ACN obtained using CAM-B3LYP-D3(BJ)/def2-TZVP level of theory. HOMO and LUMO correspond to the highest occupied molecular orbital and lowest unoccupied molecular orbital, respectively. Isovalue of 0.002 a.u. was used for visualization.

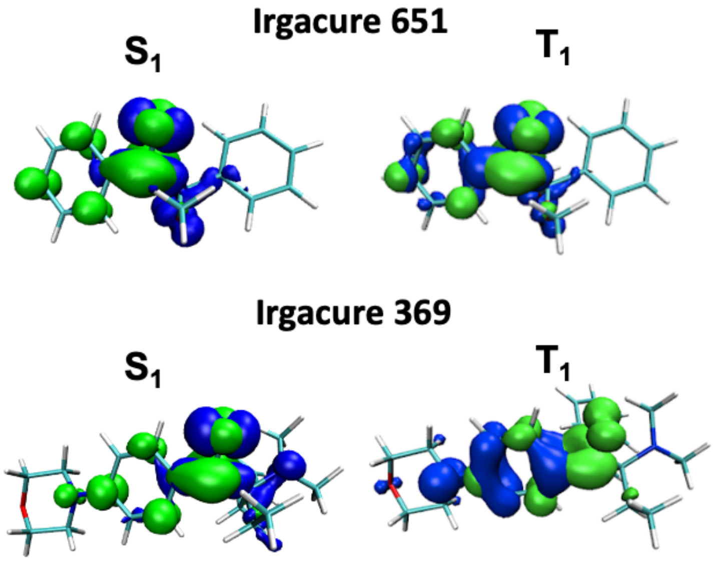

Figure S7: Visualization of electron donating (hole, in blue) and electron accepting (electron, in green) density transfer upon excitation of Irgacure 651 (top) and Irgacure 369 (bottom) from the ground state to the first singlet and triplet states in ACN. Electron-hole analysis and the visualization of the respective contributions were performed based on data obtained using TD-CAM-B3LYP-D3(BJ)/def2-TZVP level of theory. Isovalue of 0.001 a.u. was used for visualization.

The hole and electron analysis, computed in order to identify the movement of electrons upon excitation, reveals that the $S_0 \rightarrow S_1$ transition, for both Irgacure 651 and Irgacure 369, has $n\pi^*$ character (see Figure S7), involving the excitation from the non-bonding (n) to the anti-bonding (π^*) orbital localized mainly on the carbonyl group of the initiators. The T_1 state of Irgacure 651 is mainly characterized by similar $n\pi^*$ character to S_1, while for Irgacure 369 it is evident a more mixed character with $\pi\pi^*$ contribution involving the carbonyl bond of the initiator. The nature of the transitions to the first singlet excited state of Irgacure 651 and Irgacure 369 is in agreement with previously reported studies on similar systems which show $n\pi^*$ character of the first singlet state [10, 11]. The CT characteristics of the examined PIs, assessed through diverse parameters are listed in Table S13.

Table S13: For the excited states of Irgacure 651 and Irgacure 369 the charge transfer (CT) data were computed in CAM-B3LYP-D3(BJ)/def2-TZVP. H represents the average distribution of electron and hole, t indicates the degree of separation between the electron and hole in the charge transfer direction (t = D - HCT), where D represents the total magnitude of the CT length, and Sr represents the overlap of electron and hole.

**State**	**H (Å)**	**t (Å)**	**D (Å)**	**Sr (a.u.)**
		Irgacure 651		
S_1	2.110	-0.213	1.232	0.495
T_1	2.317	-1.392	0.348	0.608
		Irgacure 369		
S_1	2.346	-0.210	1.495	0.479
T_1	2.613	-0.116	1.977	0.583

A notable D index suggests a substantial distance between the regions of the hole and electron. Based on the D values, the $S_0 \rightarrow S_1$ transition of Irgacure 651, the $S_0 \rightarrow S_1$ and $S_0 \rightarrow T_1$ transitions of Irgacure 369 exhibit values greater than 1.2 Å indicating a significant CT character. In contrast, the $S_0 \rightarrow T_1$ transition of Irgacure 651 shows much lower D index, suggesting a diminished CT character. The separation of the hole and electron distribution visualized in Figure S7 show indeed that the electron and hole for the $S_0 \rightarrow S_1$ transitions of Irgacure 651 and Irgacure 369 are predominantly localized on the carbonyl bond. Although the $S_0 \rightarrow T_1$ transition of Irgacure 651 follows a similar trend, a more mixed distribution of electron and hole is observed on the phenyl ring. The $S_0 \rightarrow T_1$ transition of Irgacure 369 is characterized by a low degree of localization of the electron and hole with a higher degree of separation, resulting in a more prominent CT. The Sr indices, reflecting the overlap between the hole and electron distribution, for $S_0 \rightarrow S_1$ of Irgacure 651, $S_0 \rightarrow S_1$ and $S_0 \rightarrow T_1$ transitions of Irgacure 369 are approximately 0.5, signifying that half of the hole and electron distribution overlaps. The Sr value for the $S_0 \rightarrow T_1$ transition of Irgacure 651 is slightly higher at 0.61, indicating that more than half of the electron and hole distribution overlaps. In addition Sr in $S_0 \rightarrow T_1$ of Irgacure 651 is higher, therefore the overlap of hole and electron is higher than for $S_0 \rightarrow S_1$. The highest H index is for the $S_0 \rightarrow T_1$ transition of Irgacure 369, which has the highest CT character. The t index corresponding to the excitations $S_0 \rightarrow S_1$ of Irgacure 651 and $S_0 \rightarrow S_1$ and $S_0 \rightarrow T_1$ of Irgacure 369 is less negative than the $S_0 \rightarrow T_1$ transition of Irgacure 651, indicating a slightly higher separation of the hole and electron.

In the case of BBK, in contrast to the challenges observed for DETC[5] and Irgacure 369, the comparison of S_1 and S_0 geometries computed using both B3LYP and CAM-B3LYP (refer to Figures S8 and S9) reveals no significant divergences, which means that both DFT functionals are physically consistent for BBK and there is no unphysical structural distortion of the structure in the excited states as observed for the S_1 state of DETC.

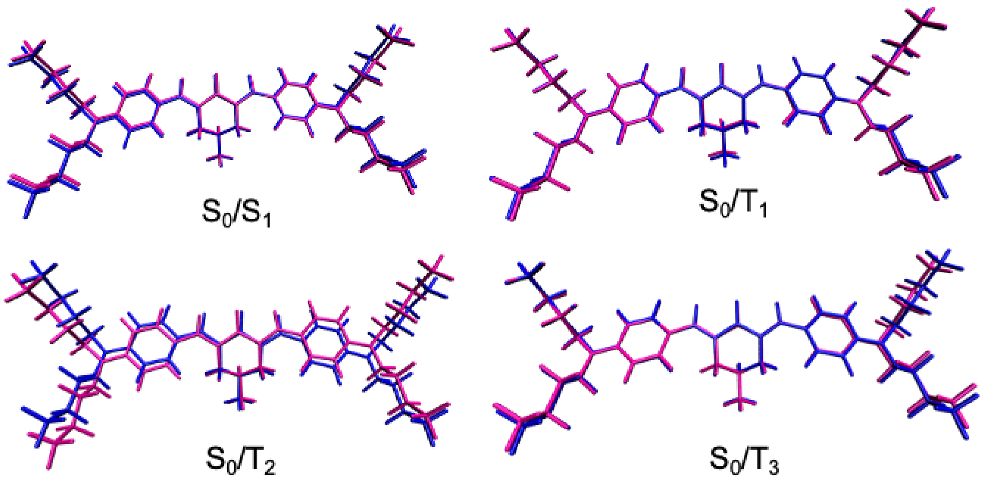

Figure S8: Graphical comparison of the ground state structure of BBK (in blue) and different excited states (in magenta). All structures were optimized with TDA-CAM-B3LYP-D3(BJ)/def2-TZVP in implicit ACN and confirmed by vibrational analysis.

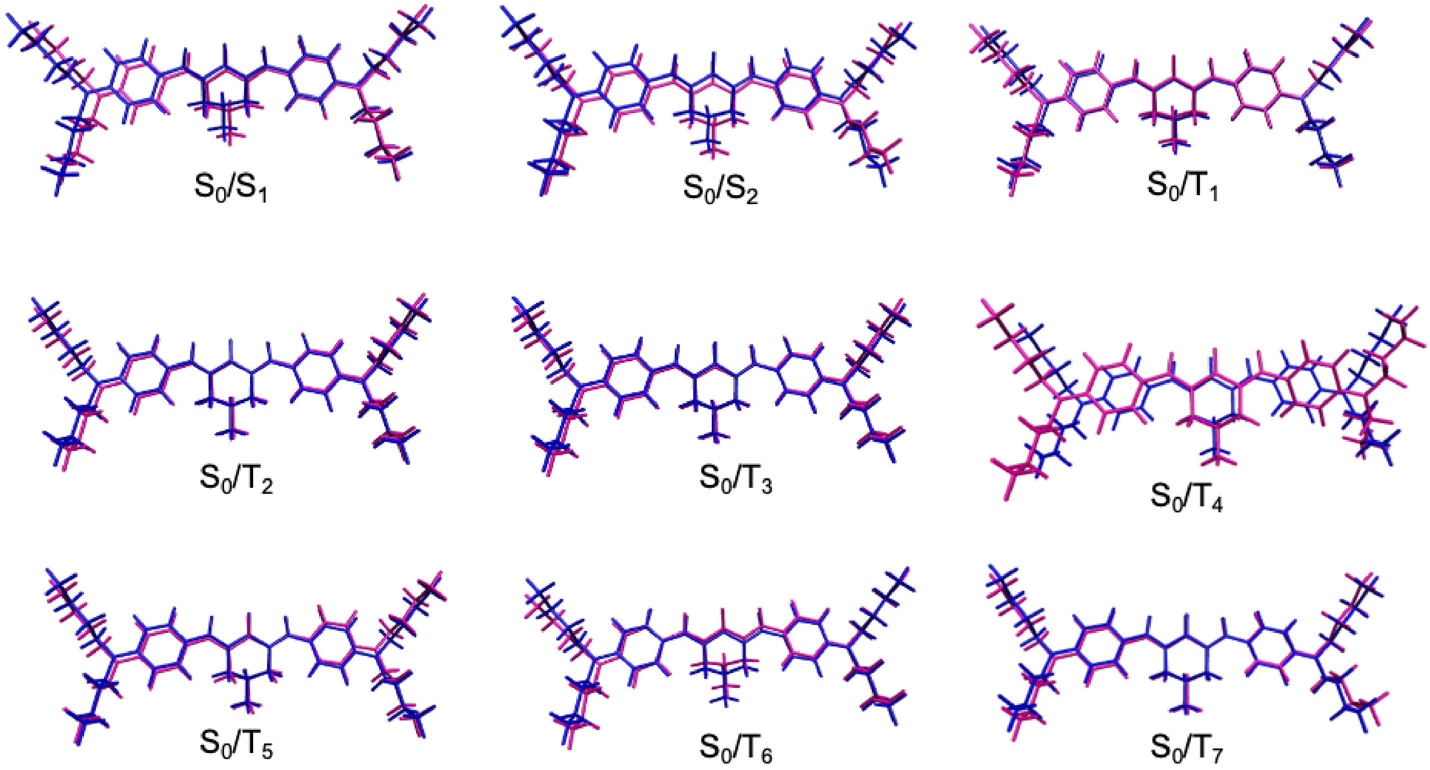

Figure S9: Graphical comparison of the ground state structure of BBK (in blue) and different excited states (in magenta). All structures were optimized with TDA-B3LYP-D3(BJ)/def2-TZVP in implicit ACN and confirmed by vibrational analysis.

Figure S10 and Table S14 show that for BBK the transition to S_1 and T_1 is mostly HOMO $\rightarrow$ LUMO delocalized all over the π system while T_3 is HOMO-1 $\rightarrow$ LUMO localized on the carbonyl bond while the CT of T_2 and high triplet states i.e. T_4-T_7 are delocalized all over the π system with T_5 and T_6 showing more pronounced localization on the phenyl rings.

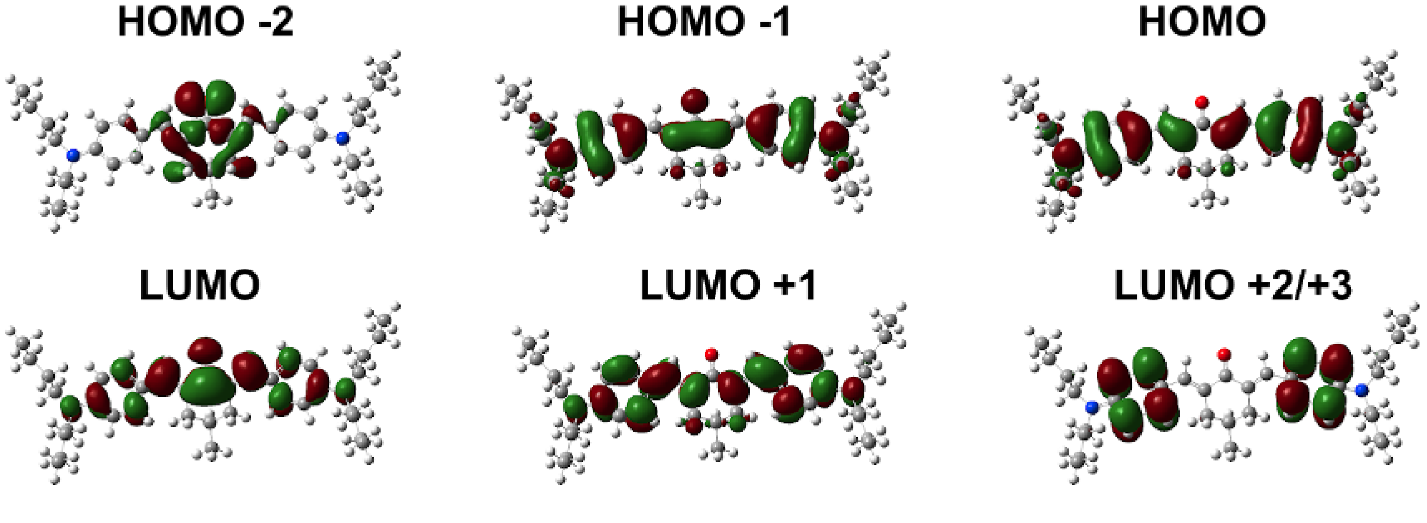

Figure S10: Visualization of molecular orbitals (MOs) of BBK in ACN obtained using CAM-B3LYP-D3(BJ)/def2-TZVP level of theory. HOMO and LUMO correspond to the Highest Occupied Molecular Orbital and Lowest Unoccupied Molecular Orbital, respectively. Isovalue of 0.002 a.u. was used for visualization. Molecular orbitals obtained for the optimized using B3LYP-D3(BJ)/def2-TZVP are similar.

Table S14: Transition orbitals of BBK in ACN with CAM-B3LYP-D3(BJ)/def2-TZVP and B3LYP-D3(BJ)/def2-TZVP with respective contributions.

**CAM-B3LYP**		
Transition	Molecular Orbitals	Contribution (%)
$S_0 \to S_1$	HOMO → LUMO	88.3
$S_0 \to S_2$	HOMO -2 → LUMO	76.9
$S_0 \to T_1$	HOMO → LUMO	71.5
$S_0 \to T_2$	HOMO -1 → LUMO	62.1
$S_0 \to T_3$	HOMO -3 → LUMO	76.2
$S_0 \to T_4$	HOMO -4 → LUMO	36.3
	HOMO → LUMO +1	22.7
$S_0 \to T_5$	HOMO → LUMO +2	52.1
$S_0 \to T_6$	HOMO → LUMO +3	51.1
$S_0 \to T_7$	HOMO -4 → LUMO	28.9
	HOMO -1 → LUMO+1	21.0
**B3LYP**		
Transition	Molecular Orbitals	Contribution (%)
$S_0 \to S_1$	HOMO → LUMO	98.4
$S_0 \to S_2$	HOMO -1 → LUMO	93.4
$S_0 \to T_1$	HOMO → LUMO	71.5
$S_0 \to T_2$	HOMO → LUMO +1	62.1
$S_0 \to T_3$	HOMO -1 → LUMO	76.2
$S_0 \to T_4$	HOMO -2 → LUMO	36.3
	HOMO → LUMO +1	22.7
$S_0 \to T_5$	HOMO → LUMO +2	52.1
$S_0 \to T_6$	HOMO → LUMO +2	60.3
$S_0 \to T_7$	HOMO → LUMO +3	58.2

Table S15: Charge transfer (CT) data of the excited states of BBK. Data were calculated with B3LYP-D3(BJ)/def2-TZVP and CAM-B3LYP-D3(BJ)/def2-TZVP in ACN. H is the average distribution of electron and hole, t is the separation degree of electron and hole in the charge transfer direction (t = D - HCT), D is the total magnitude of CT length and Sr is the overlap of electrons and holes

	**H (Å)**	**t (Å)**	**D (Å)**	**Sr (a.u.)**
		**B3LYP**		
S_1	4.998	-0.314	0.741	0.570
T_1	4.747	-0.395	0.644	0.658
		**CAM-B3LYP**		
S_1	4.630	-0.395	0.637	0.634
T_1	4.556	-0.501	0.540	0.654
T_2	4.394	-0.770	0.342	0.712
T_3	2.605	-0.559	0.566	0.423
T_4	5.029	-0.959	0.209	0.722
T_5	5.898	-1.057	0.192	0.562
T_6	5.899	-1.053	0.197	0.563
T_7	5.193	-0.820	0.318	0.723

All transitions, except T_3, are characterized by very high H values, suggesting a wide distribution of the hole and electron with low CT character, as can be seen by the hole-electron density depicted in Figures 6 and S11. The T_3 transition is characterized by a local distribution of the electron and hole. Further supporting the low CT character of BBK transitions, the D indices related to the CT lengths are lower than 0.6 Å for all transitions, with the S_1 transition showing a slightly higher mix of LE and CT character. The Sr values for BBK transitions are mostly higher than 0.5 a.u., indicating that more than half of the electron and hole overlap. An exception is T_3, which shows an Sr index of 0.4 a.u., related to its local distribution compared to the others, which are evidently wider. The t indices are all negative, indicating low CT character, with the least negative value for S_1, where a slightly higher contribution of CT character

is observed compared to the others. The same trends and observations apply to the indices computed with B3LYP related to S_1 and T_1 of BBK.

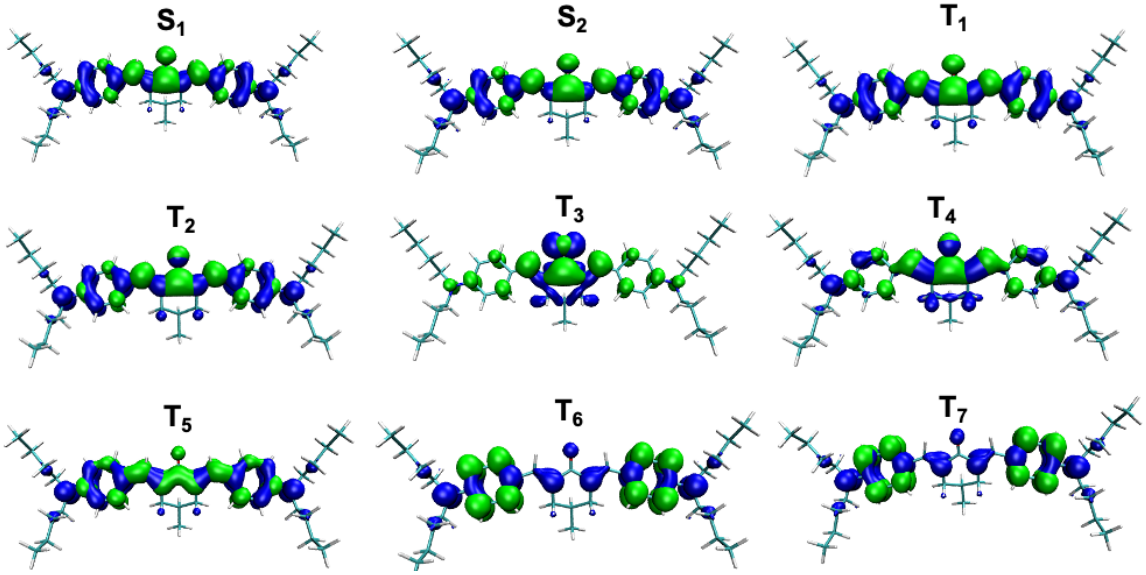

Figure S11: Visualization of electron donating (hole, in blue) and electron accepting (electron, in green) density transfer upon excitation of BBK from the ground state to singlet and triplet states in ACN. Electron-hole analysis and the visualization of the respective contributions were performed based on data obtained using TDA-B3LYP-D3(BJ)/def2-TZVP level of theory. Isovalue of 0.001 a.u. was used for visualization.

Overall, the character of the triplet transitions for BBK are characterized by a more mixed LE and CT character, with $T_1 \rightarrow T_3$ being for both mostly localized on the carbonyl bond. The maximum CT character is related to $S_0 \rightarrow S_1$ (i.e. D index is 0.64 Å) which is much lower than the one obtained for DETC[5] of 2.22 Å.

In conclusion, the discrepancy between the PIs regarding the CT representation operated by B3LYP and CAM-B3LYP might be attributed to the lower CT character of the excited states in BBK compared to DETC and Norrish type I PIs (see Table S15). B3LYP lacks indeed the correct asymptotic behavior for the electron-electron interaction, a critical aspect for precisely describing the long-range behavior of CT states. Consequently, functionals like CAM-B3LYP, characterized by range-separated and long-range corrected features, can correctly capture the electron-electron interaction. Therefore, the reliability of B3LYP functional is system dependent and, whenever CT is not present or not significant, the functional is stable in reproducing the photophysical properties of the system under investigation, with better agreement between computed and experimental absorption spectra. On the contrary, CAM-B3LYP shows repetitively a significant blue-shift (see Table S3). However, due to the inconsistency of B3LYP functional, not only for DETC but also for Irgacure 651 and Irgacure 369, CAM-B3LYP was chosen for the study of the PIs, while data computed with B3LYP are included for comparative purposes, especially in the case of BBK, in order to provide a broader overview of the photoreactivity, especially for excited states, which could not be optimized with CAM-B3LYP.

4 Two-photon absorption

Table S16: Values for two-photon absorption (2PA) as 2PA excitation energy (E, eV), 2PA wavelength (λ_{2PA}, nm), 2PA strength (δ_{2PA}, a.u.), cross section (σ_{2PA}, GM) of Irgacure 651 in ACN (PCM model) computed as reported in Computational Details. Data obtained using TD-CAM-B3LYP/def2-TZVP level of theory starting from the optimized ground state geometry. No rescaling with regard to experimental data was applied. 1 GM corresponds to 1×10^{-50} cm^4 s $photon^{-1}$ $molecule^{-1}$. The spectrum is depicted in Figure 2.

**Excitation**	**Energy**	λ_{2PA}	δ_{2PA}	σ_{2PA}
1	1.89	656.00	7.03	0.07
2	2.47	502.98	28.40	0.51
3	2.56	484.31	412.00	7.93
4	2.73	454.15	64.30	1.41
5	2.84	437.33	0.44	10.40
6	2.88	431.25	0.26	6.19
7	3.02	410.54	18.70	0.50
8	3.05	407.17	20.00	0.54
9	3.10	399.95	19.40	0.55
10	3.16	392.98	8.22	0.24

Table S17: Values for two-photon absorption (2PA) as 2PA excitation energy (E, eV), 2PA wavelength (λ_{2PA}, nm), 2PA strength (δ_{2PA}, a.u.), cross section (σ_{2PA}, GM) of Irgacure 369 in ACN (PCM model) computed as reported in Computational Details. Data obtained using TD-CAM-B3LYP/def2-TZVP level of theory starting from the optimized ground state geometry. No rescaling with regard to experimental data was applied. 1 GM corresponds to 1×10^{-50} cm^4 s $photon^{-1}$ $molecule^{-1}$. The spectrum is depicted in Figure 2.

**Excitation**	**Energy**	λ_{2PA}	δ_{2PA}	σ_{2PA}
1	2.04	609.26	238.00	2.89
2	2.11	589.00	124.00	1.61
3	2.21	562.29	4280.00	61.10
4	2.38	522.04	44.00	0.73
5	2.65	467.86	52.40	1.08
6	2.76	450.03	177.00	3.94
7	2.86	434.27	6.41	0.15
8	2.94	422.43	90.10	2.28
9	2.99	414.66	62.00	1.62
10	3.06	405.18	1050.00	28.80

Table S18: Values for two-photon absorption (2PA) as 2PA excitation energy (Energy, eV), 2PA wavelength (λ_{2PA}, nm), 2PA strength (δ_{2PA}, a.u.), 2PA cross section (σ_{2PA}, GM) of BBK in implicit ACN (PCM model). Data obtained using TDA-CAM-B3LYP/def2-TZVP level of theory and optimized ground state geometry of BBK as reported Computational details. No rescaling with regard to experimental data was applied. 1 GM corresponds to 1×10^{-50} cm^4 s $photon^{-1}$ $molecule^{-1}$.

**Excitation**	**Energy**	λ_{2PA}	δ_{2PA}	σ_{2PA}
1	1.63	760.64	2190	40.50
2	1.83	677.51	217000	1.91
3	1.91	650.83	61.10	3170.00
4	2.23	555.98	356	21.90
5	2.28	543.79	3710	57.30
6	2.55	487.17	162000	4740.00
7	2.63	471.42	687	76.40
8	2.70	460.05	753000	16000.00
9	2.72	456.66	5210	277.00
10	2.78	446.79	748	872.00

Table S19: Values for two-photon absorption (2PA) as 2PA excitation energy (Energy, eV), 2PA wavelength (λ_{2PA}, nm), 2PA strength (δ_{2PA}, a.u.), cross section (σ_{2PA}, GM) of BBK in implicit ACN (PCM model). Data obtained using TDA-B3LYP/def2-TZVP level of theory and optimized ground state geometry of BBK as reported in Computational details. No rescaling with regard to experimental data was applied. 1 GM corresponds to 1×10^{-50} cm^4 s photon^{-1} molecule^{-1}.

Excitation	Energy	λ_{2PA}	δ_{2PA}	σ_{2PA}
1	1.32	942.84	14600	74.20
2	1.54	807.71	1280000	8820.00
3	1.64	756.00	79.60	0.63
4	2.07	598.96	5740	72.10
5	2.07	598.96	25700	324.00
6	2.07	582.08	2320	30.90
7	2.14	579.36	107000	1430.00
8	2.22	558.49	1090000	15700.00
9	2.23	555.98	3200	46.50
10	2.25	551.04	32000	475.00

5 Three-photon absorption

Table S20: Values for three-photon absorption (3PA) as excitation energy (Energy, eV), wavelength (λ_{1PA}, nm), oscillator strength (Osc.str.), and 3PA as wavelength (λ_{3PA}, nm), 3PA strength (δ_{3PA}, a.u.), cross section (σ_{3PA}, cm^6 s^2 photon^{-1}) of BBK in the gas phase. Data obtained using TD-CAM-B3LYP/def2-SVP level of theory and optimized ground state geometry of BBK as reported in Computational Details.

Excitation	Energy	λ_{1PA}	λ_{3PA}	$\delta_{3PA} \times 10^8$	$\sigma_{3PA} \times 10^{-77}$
1	3.37	367.91	1103.72	4.47	1.13
2	3.44	360.42	1081.26	18.50	4.99
3	3.87	320.37	961.12	1.42	0.54
4	4.58	270.71	812.12	0.92	0.59
5	4.58	270.71	812.12	0.12	0.08
6	5.06	245.03	735.08	488	418.63
7	5.20	238.43	715.29	263	244.86
8	5.41	229.18	687.53	7.98	8.37
9	5.54	223.80	671.39	1900	2139.15
10	5.69	217.90	653.69	144	175.65

Table S21: Values for three-photon absorption (3PA) as excitation energy (Energy, eV), wavelength (λ_{1PA}, nm), oscillator strength (Osc.str.), and 3PA as wavelength (λ_{3PA}, nm), 3PA strength (δ_{3PA}, a.u.), cross section (σ_{3PA}, cm^6 s^2 photon^{-1}) of BBK in the gas phase. Data obtained using TD-B3LYP/def2-SVP level of theory and optimized ground state geometry of BBK as reported in Computational Details.

Excitation	Energy	λ_{1PA}	λ_{3PA}	$\delta_{3PA} \times 10^8$	$\sigma_{3PA} \times 10^{-77}$
1	2.97	417.45	1252.36	89.5	15.53
2	3.13	396.12	1188.35	5.11	1.04
3	3.31	374.57	1123.72	3.02	0.73
4	4.27	290.36	871.08	50.90	26.24
5	4.27	290.36	871.08	1.24	0.64
6	4.41	281.14	843.43	151.00	857.53
7	4.42	280.15	841.52	226.00	129.22
8	4.60	269.53	808.59	6.48	4.18
9	4.68	264.92	794.77	2.13	1.45
10	4.68	264.92	794.77	123.00	83.48

6 Photophysical rates

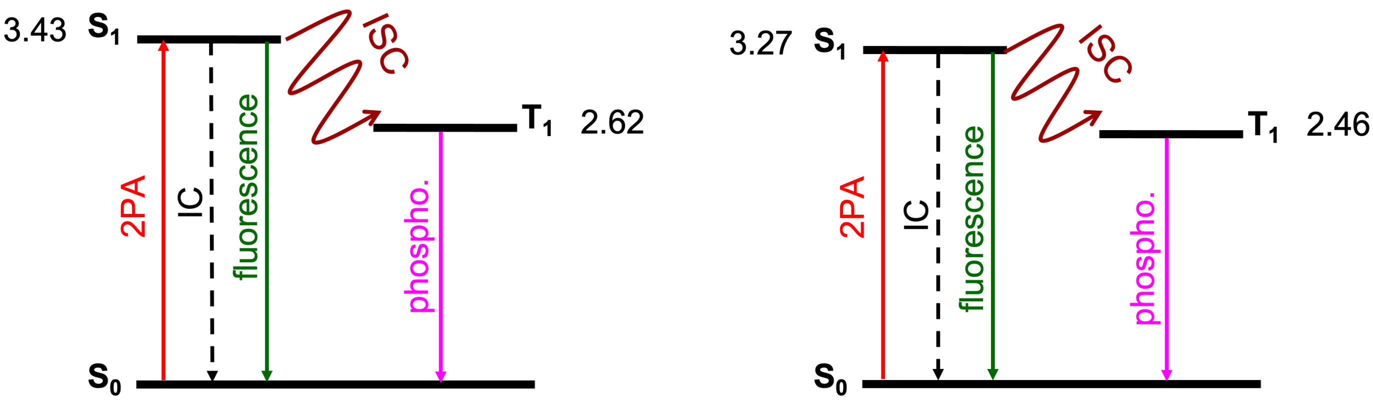

Figure S12: Jablonski diagram of Irgacure 651 (left) and Irgacure 369 (right) in TD-CAM-B3LYP-D3(BJ)/def2-TZVP in implicit ACN (PCM). Adiabatic energy values are expressed in eV and include the zero point energy correction (ZPVE).

Table S22: For Irgacure 651[a] and Irgacure 369[b]: the photophysical rates such as internal conversion (IC), intersystem crossing (ISC) and reverse ISC (RISC) computed in implicit ACN with TD-CAM-B3LYP-D3(BJ)/def2-TZVP level of theory as reported in Computational Details. The rates of internal conversion (IC) and intersystem crossing (ISC) processes involved the utilization of non-adiabatic coupling matrix elements (NACME in cm^{-1}), spin-orbit coupling (SOC in cm^{-1}), and reorganization energy (λ in eV). For radiative rates the ΔE (in eV) and lifetime (in s) are reported. All rates are calculated considering equilibrium states that are assumed to happen on the fs up to ps scale. NA refers to “not available” and in case of the rate signifies that the correlation function was not converged.

**Non-radiative rates**			
	Rate (s^{-1})	NACME (au)	λ (eV)
[a]IC $S_1 \rightarrow S_0$	1.35×10^7	0.003	0.547
[b°]IC $S_1 \rightarrow S_0$	1.37×10^7	0.003	1.222
	Rate (s^{-1})	SOC (cm^{-1})	λ (eV)
[a*]ISC $S_1 \rightarrow T_1$	4.54×10^7	1.64	0.478
[b]ISC $S_1 \rightarrow T_1$	7.92×10^7	1.20	1.232
[a*]RISC $T_1 \rightarrow S_1$	1.02×10^8	33.96	0.375
[b]RISC $T_1 \rightarrow S_1$	4.17×10^5	4.31	1.149
[**a]ISC $T_1 \rightarrow S_0$	6.56×10^5	5.87	0.763
[**b]ISC $T_1 \rightarrow S_0$	1.24×10^5	2.40	0.621
**Radiative rates**			
	Rate (s^{-1})	ΔE (eV)	lifetime (s)
[a]Fluorescence	4.53×10^5	3.43	2.21×10^{-6}
[b°]Fluorescence	4.21×10^2	3.27	0.0024
[a]Phosphorescence	7.93×10^{-1}	2.62	1.26
[b]Phosphorescence	3.96×10^{-1}	2.46	2.53

* Dushinsky rotation included. [°] S_1 in gas and S_0 in ACN combined with ΔE in ACN. [**] T_1 state lifetime is 1.53x10^{-6} s (Irgacure 651) and 8.03x10^{-6} s (Irgacure 369).

The fluorescence and IC rates for Irgacure 369 were determined using the S_1 state optimized in the gas phase due to convergence issues in the correlation function computed with MOMAP when calculating rates with S_1 in ACN (see more details in Section 6 of SI of Ref. [5]).

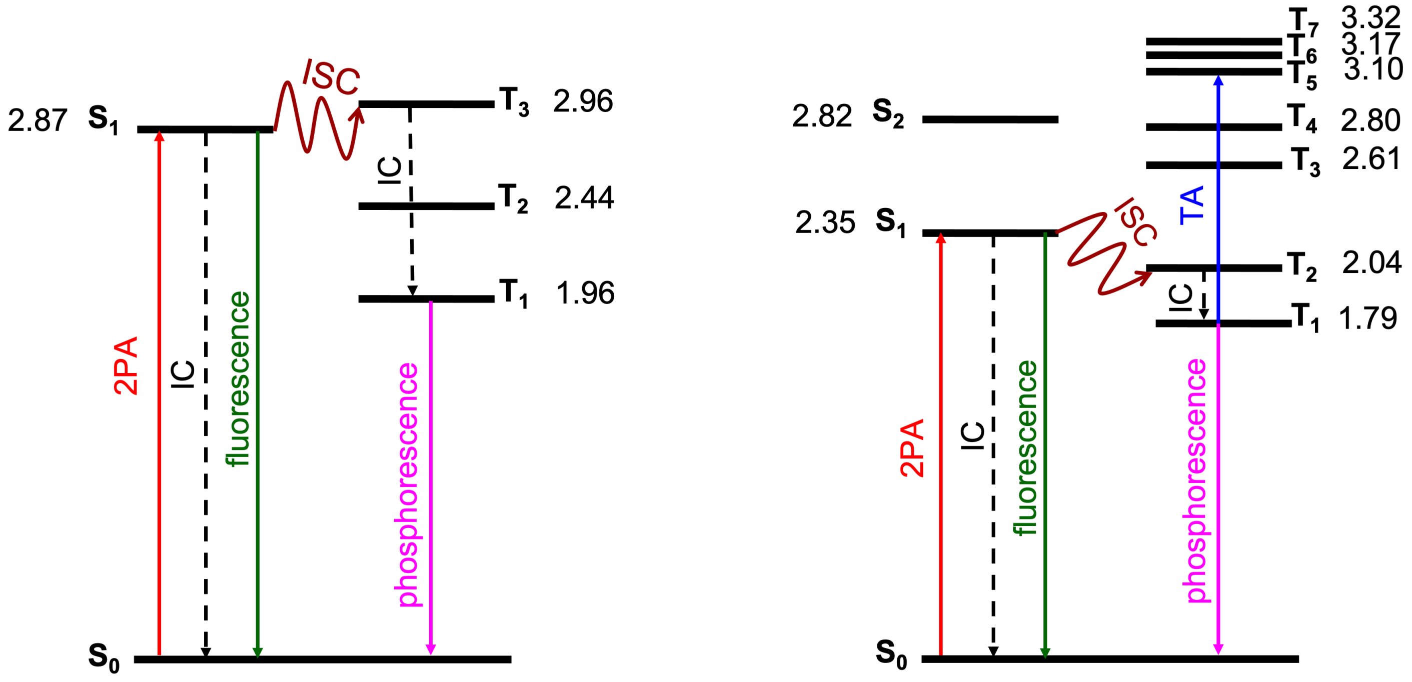

Figure S13: Jablonski diagram of BBK in TDA-CAM-B3LYP-D3(BJ)/def2-TZVP (left) and TDA-B3LYP-D3(BJ)/def2-TZVP (right) in implicit ACN (PCM). Adiabatic energy values are expressed in eV and include the zero point energy correction (ZPVE).

Table S23: Photophysical non-radiative and radiative rates for BBK in implicit ACN (PCM model) computed using TDA-CAM-B3LYP-D3(BJ)/def2-TZVP level of theory as reported in Computational Details. The rates of internal conversion (IC) and intersystem crossing (ISC) processes involved the utilization of non-adiabatic coupling matrix elements (NACME in cm^{-1}), spin-orbit coupling (SOC in cm^{-1}), and reorganization energy (λ in eV). For radiative rates the ΔE (in eV) and lifetime (in s) are reported. All rates are calculated considering equilibrium states that are assumed to happen on the fs up to ps scale. NA refers to "not available" and in case of the rate signifies that the correlation function was not converged.

**IC rates**			
Transition	Rate	NACME	λ
$S_1 \rightarrow S_0$	1.38×10^{8}	0.009	0.237
$T_3 \rightarrow T_1$	NA	0.000	0.552
$T_2 \rightarrow T_1$	8.12×10^{10}	0.005	0.326
**ISC and RISC rates**			
Transition	Rate	SOC	λ
$S_1 \rightarrow T_1$	9.68×10^{5}	0.32	0.258
$S_1 \rightarrow T_2$	1.09×10^{5}	2.83	0.076
$S_1 \rightarrow T_3$	2.12×10^{6}	3.63	0.265
$T_1 \rightarrow S_1$	3.30×10^{3}	0.20	0.287
$T_2 \rightarrow S_1$	1.59×10^{4}	1.10	0.052
$T_3 \rightarrow S_1$	2.14×10^{9}	3.35	0.255
$T_1 \rightarrow S_0$	3.64×10^{5}	1.96	0.472
**Radiative rates**			
	Rate	ΔE	lifetime
Fluorescence	6.72×10^{8}	2.87	5.81×10^{-9}
Phosphorescence	9.41×10^{-1}	1.96	1.06

Table S24: Photophysical non-radiative and radiative rates for BBK in implicit ACN (PCM model) computed using TDA-B3LYP-D3(BJ)/def2-TZVP level of theory as reported in Computational Details. The rates of internal conversion (IC) and intersystem crossing (ISC) processes involved the utilization of non-adiabatic coupling matrix elements (NACME in cm^{-1}), spin-orbit coupling (SOC in cm^{-1}), and reorganization energy (λ in eV). For radiative rates the ΔE (in eV) and lifetime (in s) are reported. All rates are calculated considering equilibrium states that are assumed to happen on the fs up to ps scale. NA refers to "not available" and in case of the rate signifies that the correlation function was not converged.

	**IC rates**		
Transition	Rate	NACME	λ
$S_1 \rightarrow S_0$	1.49×10^{8}	0.007	0.114
$S_2 \rightarrow S_0$	5.91×10^{7}	0.005	0.089
$S_2 \rightarrow S_1$	5.42×10^{11}	0.005	0.034
$T_7 \rightarrow T_1$	2.53×10^{10}	0.002	0.632
$T_6 \rightarrow T_1$	3.56×10^{11}	0.004	0.999
$T_5 \rightarrow T_1$	NA	0.002	0.731
$T_4 \rightarrow T_1$	1.19×10^{11}	0.002	0.620
$T_3 \rightarrow T_1$	1.12×10^{10}	0.002	0.247
$T_2 \rightarrow T_1$	8.12×10^{11}	0.005	0.272
	**ISC rates**		
Transition	Rate	SOC	λ
$S_1 \rightarrow T_1$	5.11×10^{5}	0.19	0.117
$S_1 \rightarrow T_2$	3.70×10^{8}	3.35	0.060
$S_1 \rightarrow T_3$	5.14×10^{6}	3.05	0.171
$*T_1 \rightarrow S_0$	8.94×10^{4}	1.96	0.182
	**RISC rates**		
Transition	Rate	SOC	λ
$T_1 \rightarrow S_1$	2.69×10^{5}	1.36	0.086
$T_2 \rightarrow S_1$	7.87×10^{5}	1.42	0.048
$T_3 \rightarrow S_1$	2.10×10^{9}	2.82	0.195
$T_4 \rightarrow S_1$	NA	0.75	NA
$T_5 \rightarrow S_1$	4.72×10^{7}	3.46	0.186
$T_6 \rightarrow S_1$	1.65×10^{7}	1.82	0.262
$T_7 \rightarrow S_1$	NA	0.22	0.263
$T_3 \rightarrow S_2$	6.23×10^{4}	0.36	0.214
$T_4 \rightarrow S_2$	NA	1.33	NA
$T_5 \rightarrow S_2$	5.21×10^{8}	1.38	0.356
$T_6 \rightarrow S_2$	1.81×10^{6}	0.10	0.579
$T_7 \rightarrow S_2$	5.93×10^{7}	1.40	0.133
	**Radiative rates**		
	Rate	ΔE	lifetime
Fluorescence	4.15×10^{8}	2.35	2.41×10^{-9}
Phosphorescence	1.15×10^{0}	1.79	0.869

* lifetime of T_1 is 1.12x10^{-5} s

6.1 Investigation of the reaction cleavage for Norrish type I PIs

Results listed in Table S25 show that for Irgacure 369 the T_1 energy is higher than the bond energy in the case of β-cleavage (with both DFT functionals) but not in the case of α-cleavage with CAM-B3LYP for Irgacure 369. However, due to the almost negligible difference between the T_1 energy and the bond energy in the latter case (only 0.07 eV) and considering that the T_1 energy is higher than the bond energy considering B3LYP, it is possible to state that the dissociation reaction will occur.

Table S25: Comparison of bond dissociation energy (BDE) as (E_{BOND}) and first triplet excited state energy (E_{T_1}) for Irgacure 651 and Irgacure 369.

		E_{BOND}		E_{T_1}		$E_{T_1} > E_{BOND}$
	cleavage	eV	kcal mol^{-1}	eV	kcal mol^{-1}	eV
			CAM-B3LYP			
Irgacure 651	α-	2.48	55.20	2.62	60.30	2.62 >2.48
Irgacure 369	α-	2.49	57.46	2.42	56.56	2.42 <2.49
	β-	2.08	47.85			2.42 >2.08
			B3LYP			
Irgacure 651	α-	2.48	57.39	2.62	60.34	2.62 >2.48
Irgacure 369	α-	2.42	56.02	2.46	55.68	2.46 >2.42
	β-	2.00	46.12			2.46 >2.00

Table S26: Electronic spin density values on the radical fragments (in a.u.) related to the α- and β-cleavage of Irgacure 651 and Irgacure 369 computed with CAM-B3LYP-D3(BJ)/def2-TZVP and B3LYP-D3(BJ)/def2-TZVP in ACN.

**Photoinitiator**	**cleavage**	**atoms**	**spin density**
	CAM-B3LYP		
Irgacure 651	α-cleavage	C12	0.66
		C13	0.82
Irgacure 369	α-cleavage	C27	0.84
		C25	0.75
	β-cleavage	N39	0.92
		C27	0.92
	B3LYP		
Irgacure 651	α-cleavage	C12	0.65
		C13	0.79
Irgacure 369	α-cleavage	C27	0.82
		C25	0.66
	β-cleavage	N39	0.91
		C27	0.92

BBK shows that the energy of T_1 is lower than the bond energy (E_{BOND}), typical feature for Norrish type II PIs that do not generate radicals upon cleavage of the first triplet state (see Table S27). Atoms numbering for BBK is reported in FigureS2.

Table S27: Comparison of the bond dissociation energy (E_{BOND}) with the first triplet excited state optimized energy (E_{T1}) for BBK computed in CAM-B3LYP-D3(BJ)/def2-TZVP in ACN. For efficient bond scission from the triplet state, the triplet energy must be higher than the bond energy.

BOND	E_{BOND} eV	E_{BOND} kcal mol^{-1}	E_{T1} eV	E_{T1} kcal mol^{-1}	$E_{T1} < E_{BOND}$ eV
C16-C17	5.08	117.23	2.34	53.93	2.34 <5.05
C35-C36	4.00	92.39			2.34 <4.00
C12-C15	3.70	85.23			2.34 <3.70
C34-C35	2.96	68.31			2.34 <2.96
C33-C34	3.24	74.70			2.34 <3.24
C45-N26	2.22	51.25			2.34 >2.22

6.2 Radical formation mechanisms of BBK

6.2.1 Triplet absorption

Table S28: Triplet vertical excitation energies (in eV and nm) of BBK computed using (U)B3LYP-D3(BJ)/def2-TZVP and (U)CAM-B3LYP-D3(BJ)/def2-TZVP in ACN starting from the optimized first triplet state. To note that the transitions reported in the Table are not directly related to triplet excited states numbering used in Table S3 but further analysis is necessary to identify the nature of the triplet states.

	**B3LYP**			**CAM-B3LYP**		
Excitation	Energy	Wavelength	Osc.str.	Energy	Wavelength	Osc.str.
1	0.70	1777.83	0.0679	1.71	724.17	0.0068
2	1.20	1033.81	0.0007	1.93	642.98	0.0074
3	1.67	743.34	0.0037	1.95	636.25	0.2921
4	1.95	635.82	1.6025	2.38	521.72	0.9421
5	2.07	599.67	0.0717	2.70	459.61	0.0344
6	2.11	587.3	0.0017	3.05	406.04	0.1910
7	2.41	514.09	0.0029	3.10	400.03	0.0066
8	2.49	497.01	0.3286	3.48	356.51	0.0186
9	2.63	471.98	0.0003	3.64	340.77	0.0024
10	2.67	463.95	0.0085	3.79	326.91	0.1075

6.2.2 Formation of radicals

Table S29: Gibbs free energies (in kcal mol^{-1}) related to the Norrish type II radical formation reactions through the inter-HAT mechanism between BBK, in both the S_0 state and different excited states, with PETA (in R_1). All calculations were performed at the (U)B3LYP-D3BJ/def2-TZVP level of theory in implicit ACN.

**State of BBK**	**PETA** R_1
S_0	48.83
S_1	-3.46
T_1	7.64
T_2	1.59
T_3	-11.56
T_4	-16.73
T_5	-22.02
T_6	-23.37
T_7	-26.99

Table S30: Gibbs free energies (in kcal mol^{-1}) related to photoactivated H-abstraction mechanism for BBK either in S_0 and in different excited states. The change in the Gibbs free energies of reaction were computed as reported in the main body. All calculations were performed in implicit ACN with (U)CAM-B3LYP-D3BJ/def2-TZVP and (U)B3LYP-D3BJ/def2-TZVP level of theory. The representation of the H atoms abstracted from the molecule is reported in Figure S14.

**State**	H_3	H_4	H_2	H_1
CAM-B3LYP				
S_0	94.60	88.81	94.14	98.09
S_1	27.93	22.15	27.48	31.42
T_1	49.69	43.91	49.24	53.18
T_2	38.02	32.24	37.56	41.51
T_3	26.33	20.55	25.88	29.82
B3LYP				
S_0	92.18	85.18	92.49	96.68
S_1	39.90	32.89	40.20	44.39
T_1	50.99	43.98	51.30	55.48
T_2	44.95	37.94	45.25	49.44
T_3	31.79	24.78	32.10	36.29
T_4	26.62	19.61	26.93	31.12
T_5	21.33	14.32	21.64	25.83
T_6	19.98	12.97	20.29	24.48

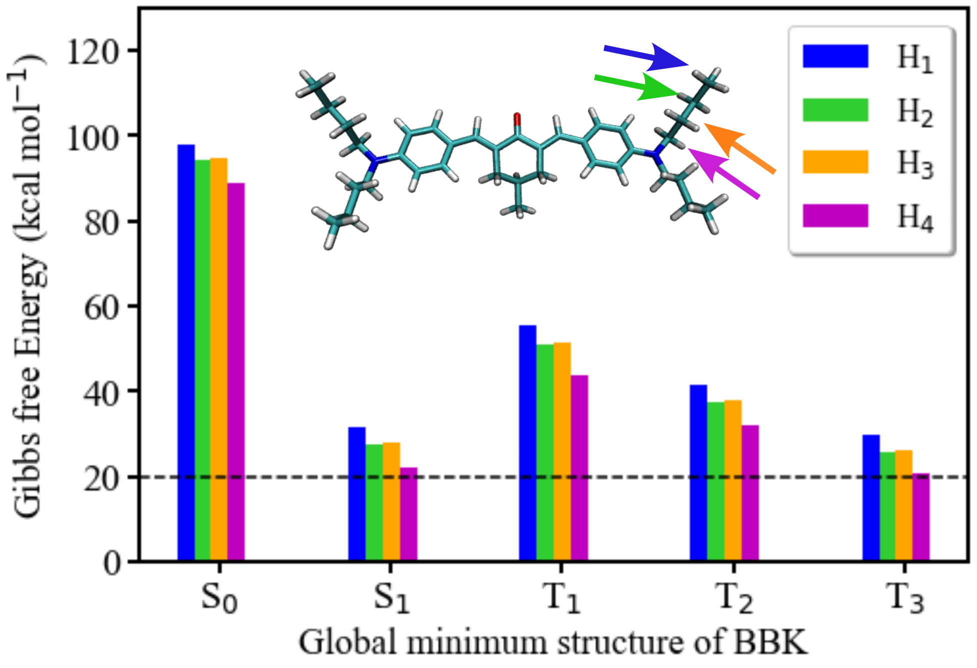

Figure S14: Gibbs free energies (in kcal mol^{-1}) related to photoactivated H-abstraction mechanism for BBK either in S_0 and in different excited states.

Table S31: Gibbs free energies (in kcal mol^{-1}) for the photolysis of BBK. Bonds are depicted in Figure 9 and S2. All energies were calculated with (U)CAM-B3LYP-D3(BJ)/def2-TZVP and (U)B3LYP-D3(BJ)/def2-TZVP level of theory in implicit ACN.

**State**	**C16-C17**	**C35-C36**	**C12-C15**	**C34-C35**	**C33-C34**	**C45-N26**
			CAM-B3LYP			
S_0	102.73	77.29	70.78	68.31	58.94	51.25
S_1	36.06	10.62	4.11	1.64	-7.73	-15.42
T_1	57.82	32.38	25.87	23.40	14.02	6.34
T_2	46.15	20.71	14.20	11.74	2.35	-5.34
T_3	34.46	9.02	2.51	0.04	-9.33	-17.02
			B3LYP			
S_0	102.83	76.63	69.63	66.90	57.69	49.75
S_1	50.54	24.34	17.34	14.61	5.41	-2.53
T_1	61.64	35.44	28.43	25.71	16.50	8.56
T_2	55.59	29.39	22.39	19.66	10.45	2.51
T_3	42.44	16.23	9.24	6.51	-2.70	-10.64
T_4	37.27	11.06	4.06	1.33	-7.87	-15.81
T_5	31.98	5.78	-1.22	-3.95	-13.16	-21.10
T_6	30.63	4.43	-2.57	-5.30	-14.50	-22.45
T_7	27.01	0.80	-6.20	-8.92	-18.13	-26.07

References

[1] H. Kaczmarek, P. Galka, *TOCPCJ* **2008**, *1*, 1 8.

[2] K. J. Schafer, J. M. Hales, M. Balu, K. D. Belfield, E. W. Van Stryland, D. J. Hagan, *Journal of Photochemistry and Photobiology A: Chemistry* **2004**, *162*, 2-3 497.

[3] L. van Lith, **2016** URL `https://api.semanticscholar.org/CorpusID:55811495`.

[4] P. Somers, Z. Liang, J. E. Johnson, B. W. Boudouris, L. Pan, X. Xu, *Light Sci Appl* **2021**, *10*, 1 199.

[5] A. Mauri, P. Kiefer, P. Neidinger, T. Messer, N. M. Bojanowski, L. Yang, S. Walden, A.-N. Unterreiner, C. Barner-Kowollik, M. Wegener, W. Wenzel, M. Kozlowska, *Chem. Sci. 15*, 32 12695.

[6] A. D. Becke, *The Journal of Chemical Physics* **1993**, *98*, 7 5648.

[7] C. Faber, P. Boulanger, C. Attaccalite, I. Duchemin, X. Blase, *Phil. Trans. R. Soc. A.* **2014**, *372*, 2011 20130271.

[8] F. J. Avila Ferrer, J. Cerezo, E. Stendardo, R. Improta, F. Santoro, *J. Chem. Theory Comput.* **2013**, *9*, 4 2072.

[9] D. Jacquemin, A. Planchat, C. Adamo, B. Mennucci, *Journal of Chemical Theory and Computation* **2012**, *8*, 7 2359.

[10] N. M. Bojanowski, A. Vranic, V. Hahn, P. Rietz, T. Messer, J. Brückel, C. Barner-Kowollik, E. Blasco, S. Bräse, M. Wegener, *Adv Funct Materials* **2022**, 2212482.

[11] E. G. Leggesse, W.-R. Tong, S. Nachimuthu, T.-Y. Chen, J.-C. Jiang, *Journal of Photochemistry and Photobiology A: Chemistry* **2017**, *347* 78.
